# Necroptosis is Related to Anti-PD-1 Treatment Response and Influences the Tumor Microenvironment in Head and Neck Squamous Cell Carcinoma

**DOI:** 10.3389/fgene.2022.862143

**Published:** 2022-05-25

**Authors:** Qiwei Wang, Fang Wang, Yinan Zhao, Guolin Tan

**Affiliations:** ^1^ Department Otolaryngology Head and Neck Surgery, Third Xiangya Hospital, Central South University, Changsha, China; ^2^ Otorhinolaryngology, Klinikum Rechts der Isar of the Technical University of Munich, Munich, Germany; ^3^ Xiangya School of Nursing, Central South University, Changsha, China

**Keywords:** necroptosis, PD1 = programmed cell death protein 1, the tumor immune microenvironment, head and necek squamous cell carcinoma, immunephenotype

## Abstract

The latest research suggesting that necroptosis plays a vital role in immune response. However, the influence of necroptosis on tumor microenvironment (TME) remodeling and immunotherapy is still unclear. We analyzed the variations in the expression of 26 necroptosis-related molecules in HNSCC and the influence of genome changes. We investigated HNSCC samples and determined that there are two necroptosis phenotypes in HNSCC cancer, and there are significant differences in cell infiltration characteristics and survival in different necroptosis phenotypes. We used the single‐sample gene set enrichment analysis to measure the level of necroptosis in patients with NecroticScore, we confirmed that the NecroticScore can predict the prognosis of HNSCC patients and the response to immunotherapy. Patients with a high NecroticScore are more sensitive to immunotherapy and have a better prognosis. Our study suggests a significant correlation between the expression imbalance of necroptosis-related molecules and suggests necroptosis plays an important role in modeling the TME. In addition, we construct a risk prediction model which could stratify patients with HNSCC and predict patient prognosis according to this necroptosis-related molecules. In conclusion, evaluating necroptosis modification patterns contributes to enhancing our understanding of TME and can guide more effective immunotherapy strategies.

## Introduction

Head and neck squamous cell carcinoma morbidity was the seventh highest in the world in 2018 (890,000 new cases and 450,000 deaths) (Bray et al., 2018). HNSCC has a high rate of metastasis and recurrence is likely due to the interactions of immune cells infiltration which make up the tumor microenvironment (TME). Studies proved that the tumor microenvironment plays a key role in immunotherapy responsiveness (Curran et al., 2010). Although the immunological therapy of the programmed death 1 (PD-1) immune-checkpoint inhibitors has greatly influenced the treatment of squamous-cell carcinoma of the head and neck, there are some HNSCC patients who benefit from anti–PD-1 antibodies therapy (Chow, 2020). Considering the economic burden for patients and the individual heterogeneity of the HNSCC tumor, it is necessary to develop comprehensive therapy targets and economical treatment plans.

Necroptosis is a caspase-independent death program. Necroptosis is triggered by toll-like receptor 3 and 4 agonists, tumor necrosis factor, certain viral infections, the T cell receptor, and the compromised activity of the protease caspase-8 (Newton and Manning, 2016). In recent research, intervening necroptosis-related molecules such as receptor-interacting protein kinase 3 (RIPK3) will enhance the response to immunotherapy (Um et al., 2020). In addition, there was a significantly positively correlation between pMLKL status and some immune signatures like PD-L1 expression. Patients with high pMLKL-positive expression were significantly associated with an increased copiousness of CD8+  T cell infiltration and a longer overall survival (OS) (Lomphithak et al., 2021). Moreover, Yatim N. et al. (Yatim et al., 2015) found that the necroptosis process was associated with NF-κB signaling, which activated CD8+ T cells to kill tumor cells. These studies have profoundly expanded our comprehension of the mechanism of necroptosis-related molecules anti-tumor effect.

However, previous research focused only on one or two necroptosis-related molecules; the necroptosis process and immune therapy reaction are the effects from factors interaction with complex network. Presently, there is no integrated study on HNSCC cancer immunity and HNSCC tumor microenvironment of necroptosis-related molecules, so the regulation mechanism of necroptosis in HNSCC cancer is still indistinct. Therefore, it is necessary to analyze the necroptosis-related gene expression pattern of HNSCC cancer and to explore the relationship between numerous necroptosis-related molecules and immune infiltration in the tumor microenvironment. In our study, we detected the expression heterogeneity of 26 necroptosis-related genes in HNSCC cancer. We concentrated on discovering the potential advantages of immunotherapy for treating HNSCC cancer. We integrated these necroptosis-related genes with HNSCC immunity and tumor microenvironment. Our results showed that necroptosis exerts crucial influence on the tumor immune infiltration. We established a system to evaluate this called NecroticScore, which measures the expression rate of necrotic molecules and immune status in HNSCC. We validated that the NecroticScore is a reliable prognostic value for HNSCC cancer and could guide immunotherapy treatment. We also constructed and validated necroptosis-related molecules prognostic signatures that may help to predict individual odds of death and help clinicians manage patients with HNSCC cancer.

## Methods and Materials

The workflow of our research included TCGA database and GEO database (GSE65858). We collected 26 necroptosis-related genes from previous studies (Frank and Vince, 2019; Robinson et al., 2019; Tonnus et al., 2021). Based on the expression level of 26 necroptosis-related genes, we used “ClassDiscovery” package in R to analyze datasets (Qin et al., 2020). Single‐sample gene set enrichment analysis (ssGSEA) in R package GSVA was used to construct a system to evaluate the score of the expression of 26 necroptosis-related genes in HNSCC patient. This system was named NecroticScore. We validated distribution differences of NecroticScore in HNSCC and further valuated the predictive capacity of NecroticScore for the prognosis and immunotherapy effect in HNSCC patients. The pathway enrichment analysis of this necroptosis-related molecules was performed with package “clusterProfiler” (Yu et al., 2012). HNSCC cell line CAL-27 and normal nasopharyngeal epithelial cell line (NP69) were used to assay quantitative real-time PCR. All statistical analyses used R (https://www.r-project.org/).

## Result

### Enrichment Functions Analysis in 26 Necroptosis-Related Molecules

**FIGURE 1:**
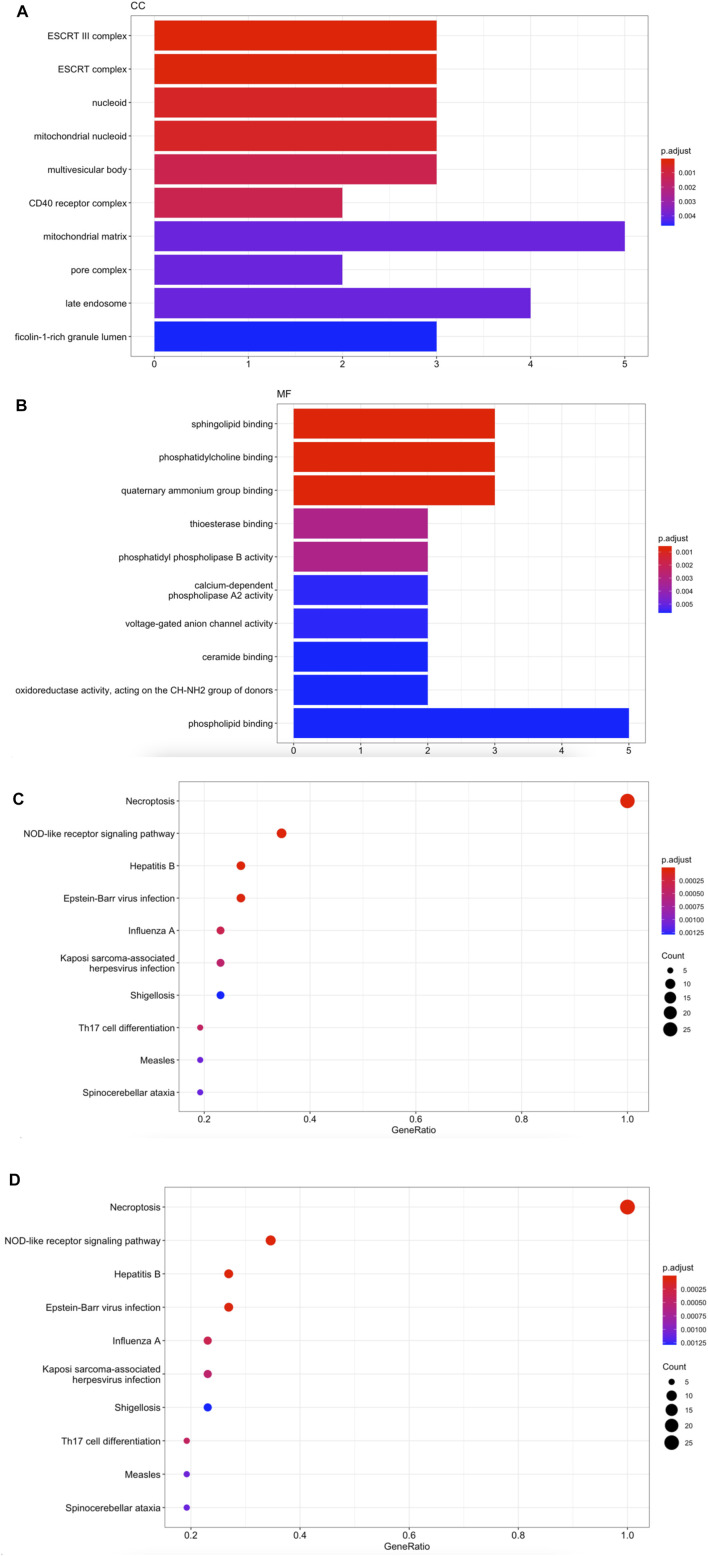
Enrichment functions analysis in 26 necroptosis-related molecules. **(A)** CC analysis for 26 necroptosis-related genes. **(B)** MF analysis for 26 necroptosis-related genes. **(C)** BP analysis for 26 necroptosis-related genes. **(D)** KEGG enrichment analysis for 26 necroptosis-related genes.

We collected 26 necroptosis-related molecules from existing studies (Frank and Vince, 2019; Robinson et al., 2019; Tonnus et al., 2021). The 26 molecules are HSP90AA1, SLC25A5, CAPN1, TYK2, VDAC2, CHMP3, CHMP1B, PYGL, STAT6, VDAC1 MACROH2A1, GLUD1, CHMP1A, GLUD2, PLA2G4B, IFNA21, TICAM2, TRAF2, PPID, MAPK9, ZBP1, PLA2G4C, IRF9, TLR3, TRAF5, and JAK3. We used GO analysis to reveal that necroptosis-related genes were mainly enriched in actions such as ESCRT complex, phosphatidylcholine binding, programed necrotic cell death, and necroptotic process (Figures 1A–C). KEGG enrichment analysis validated that 26 necroptosis-related genes were associated with Necroptosis and NOD-like receptor signaling pathway (Figure 1D).

### Transcriptome Analysis Revealed Necroptosis is Associated With Survival in HNSCC

**FIGURE 2:**
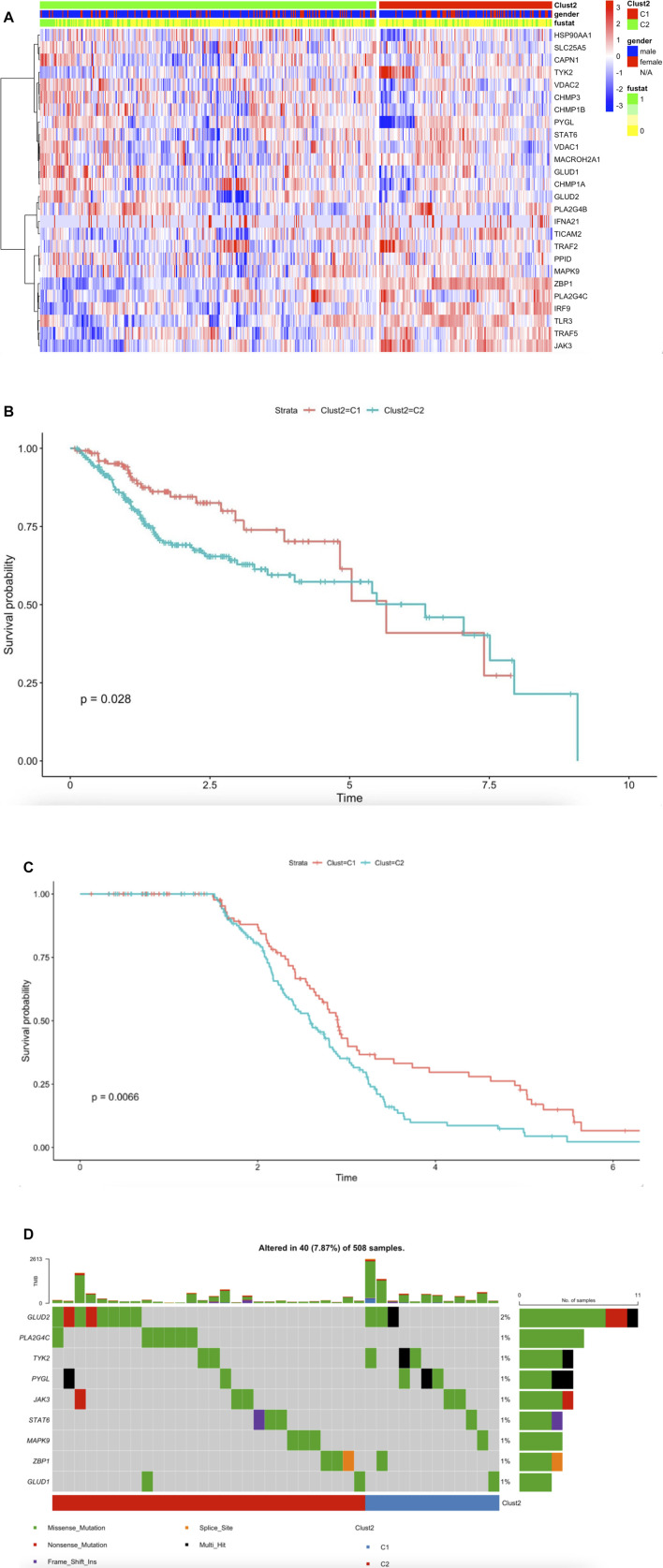
Transcriptome analysis revealed necroptosis is associated with survival in HNSCC. **(A)** The synthetic thermogram displays the correlation between the two types of necroptosis and the expression variance of the 26 necroptosis-related molecules (Meta-cohort), C1 (170cases), C2 (331cases), “1” means dead, “0” means alive, “fustat” means survival status. **(B)** The Kaplan-Meier plot displays significant differences of survival rate among the two kinds of necroptosis phenotypes in the TCGA database. C1 (131cases) was better than C2 (253cases). Unit of Time (years). **(C)** The GSE65858 Kaplan-Meier plot displays significant differences of survival rate among the two kinds of necroptosis phenotypes. C1 (113cases) was better than C2 (154cases). Unit of Time (years). **(D)** The waterfall plot displays the nine necroptosis molecules somatic mutations with mutation frequency in HNSCC.

To further understand the mechanism of necroptosis regulator integration in HNSCC cancer, we collected a total of 501 head and neck squamous cell carcinoma samples from The Cancer Genome Atlas (TCGA) database (Supplementary Table1). We identified two unique modification patterns by using the R package “ClassDiscovery”, named Clust2_C1 (170 cases), Clust2_C2 (331 cases). We used thermogram to analyze expression level of necroptosis genes. We found necroptosis molecules in Clust2_C1 had a higher expression level compared to Clust2_C2 (Figure 2A). We used HNSCC-TGCA survival cohort to analyze the different curves in survival model between the two subtypes of necroptosis; we found Clust2_C1 (131cases) OS provides a particularly significant survival advantage while Clust2_C2 (253cases) has poor prognosis (Figure 2B) (log-rank, *p* = 0.028). According to the necroptosis-related genes, we analyzed GSE65858 dataset by the same method of R package, dividing 267 patients into Clust_C1 (113cases) and Clust_C2 (154cases) stratifications. The Kaplan-Meier curve (OS) also displayed a significant difference of survival proportion among the two kinds of necroptosis phenotypes (log-rank, *p* = 0.0066) (Figure 2C). This result suggested that poor prognosis for patients may be linked to low expression of necroptosis-related genes. Tumor mutational burden (TMB) was considered a promising indicator for good prognosis (Chalmers et al., 2017). We displayed the somatic mutations of the nine necroptosis genes with the highest mutation frequency in HNSCC by a waterfall diagram, but we found there was no significant differences in these genes among the two clusters (Figure 2D).

### NecroticScore is a Predictive Factor for Survival and Stratifies the Immunophenotype and HPV Status

**FIGURE 3:**
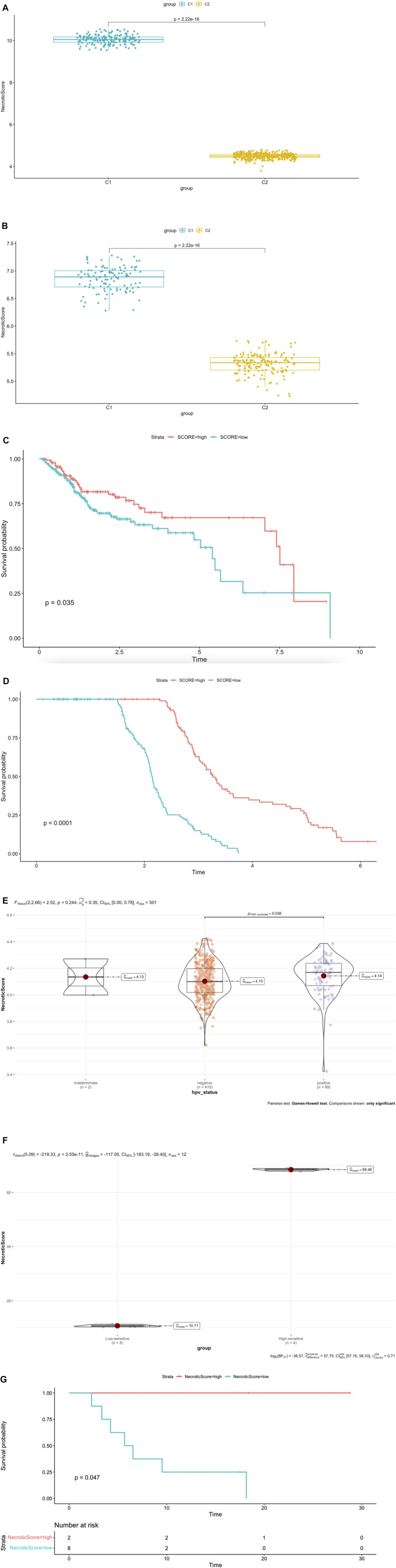
NecroticScore is a predictive factor for survival and stratifies the immunophenotype and HPV status. **(A–B)** NecroticScore in TCGA and GSE65858 dataset. **(C)** The Kaplan-Meier plot displays significant differences of survival rate among the high-NecroticScore and low-NecroticScore in the TCGA database, high (140cases), low (244cases), Unit of Time (years). **(D)** The Kaplan-Meier plot displays significant differences of survival rate among the high-NecroticScore and low-NecroticScore in the GSE65858 database, high (114cases), low (153cases) Unit of Time (years). **(E)** NecroticScore in TCGA dataset among the group of HPV (+) and HPV (−). **(F)** NecroticScore in Low-sensitive and High-sensitive group from external immunotherapy cohort (*p* value =2.55e-11). **(G)** The Kaplan-Meier plot exhibited a significant statistic *p* value of overall survival rate among the two NecroticScore groups in the external immunotherapy cohort. Unit of Time (months).

The single-sample gene set enrichment analysis (ssGSEA) in R package GSVA was used to construct a system to evaluate the score which represents the levels of necroptosis-related genes in both TCGA dataset and GSE65858 dataset; we named it NecroticScore. We found C1 NecroticScore was significantly higher than C2 in both TCGA and GSE65858 datasets (Figures 3A–B). In addition, our results show that the NecroticScore can effectively classify necroptosis phenotypes in HNSCC cancer patients. We summarized 384 patients in the TCGA cohort according to their NecroticScore and divided it into high or low group by method of the optimal cut-off value from the R package “Survminer”; we found NecroticScore was a prognostic factor to head and neck squamous cell carcinoma (log-rank, *p* = 0.035, Figure 3C). To further verify this, we plotted a Kaplan-Meier curve to observe the correlation between the NecroticScore and the stratification of HNSCC patients in the GSE65858 database. The result also showed NecroticScore was a prognostic factor to GEO cohort (log-rank, *p* < 0.0001, Figure 3D). In addition, our study revealed the patients with HPV (+) HNSCC have better overall survival than HPV (−) HNSCC (Ang et al., 2010); interestingly, we found NecroticScore in group HPV (+) was higher than HPV (−) in TCGA cohort (*p* = 0.036; Figure 3E). In the external immunotherapy cohort (Yang S. Y. C. et al., 2021), the group of high-sensitive to immune checkpoint inhibitors (ICIs) response had a significantly higher NecroticScore than the low sensitive group (*p* = 2.55e-11, Figure 3F) by the optimal cut-off value. We also found the high NecroticScore group had a longer survival time (log-rank, *p* = 0.047; Figure 3G).

### NecroticScore Influences the Tumor Immune Microenvironment in HNSCC

**FIGURE 4:**
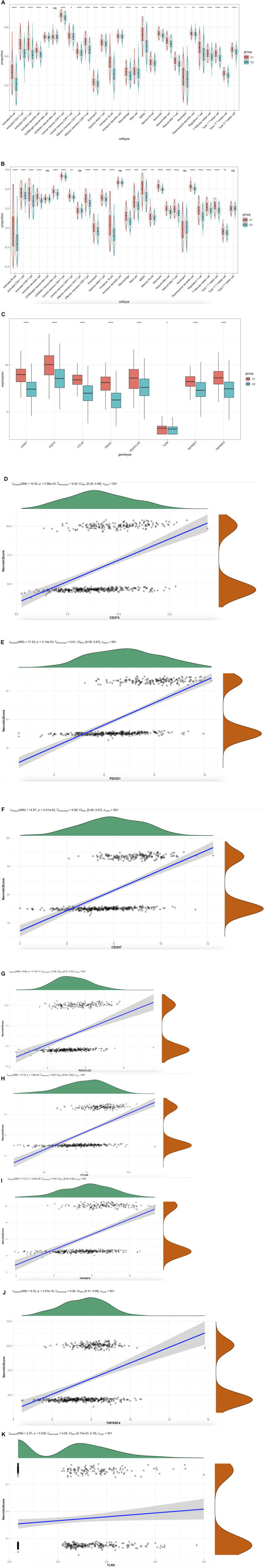
NecroticScore influences the tumor immune microenvironment in HNSCC. **(A)** Enrichment of each immune cell type infiltrating in high-score (C1) and low-score (C2) groups; from TCGA; the asterisk represents the different *p* values (* < 0.05; ** < 0.01; *** < 0.001, **** < 0.0001), C1 (170case), C2 (331cases). **(B)** Enrichment of each immune cell type infiltrating in high-score (C1) and low-score (C2) groups; from GSE65858; the asterisk represents the different *p* values (* < 0.05; ** < 0.01; *** < 0.001, **** < 0.0001), C1 (115cases), C2 (155cases). **(C)** Differential expression of Immune checkpoint genes in C1 and C2 groups; from TCGA cohort; the asterisk represents the different *p* values (* < 0.05; ** < 0.01; *** < 0.001, **** < 0.0001), C1 (170case), C2 (331cases). **(D–K)** Correlation between NecroticScore and CD274, PDCD1, CD247, PDCD1LG2, CTLA4, TNFRSF9, TNFRSF4, and TLR9.

To detect the relationship between the immune infiltration outlook of multi-immune cell types and NecroticScore, we divided tumor samples from TCGA dataset into two group. We calculated the relative immune cell infiltration levels of single sample by R package GSVA, and used gene signatures expressed by immune cell populations to individual cancer samples (Bindea et al., 2013; Barbie et al., 2009). We found the infiltration levels of the C1 group were significantly higher than the C2 group (Figure 4A). Similarly, we also found a significant difference between groups of immune infiltration level in GSE65858 database (Figure 4B). Considering the HNSCC individual variability and complexity for anti-PD-L1-treatment, we used NecroticScore to explore the mechanism of the effect of it on immune checkpoint inhibitors in head and neck squamous cell carcinoma. We summarized data on eight related immune checkpoint genes (CD247, CD274, PDCD1, PDCD1LG2, TNFRSF9, TNFRSF4, CTLA4, and TLR9) from existing studies (Ramos-Casals et al., 2020; Huang et al., 2021; van de Donk et al., 2021), and found they have significant differential expression between high and low NecroticScore group (Figure 4C). We also further detected the correlation between NecroticScore and eight related immune checkpoint genes expression. We found NecroticScore was positively correlated with eight Immune checkpoint genes expression (Figures 4D–K) (CD274: r = 0,42, *p* = 2.98e-23; PDCD1: r = 0.61, *p* = 4.14e-53; CD247: r = 0.56, *p* = 4.01e-42; PDCD1LG2: r = 0.30, *p* = 1.17e-11; CTLA4: r = 0.56, *p* = 1.06e-42; TNFRSF9: r = 0.45, *p* = 2.04e-26; TNFRSF4: r = 0.39, *p* = 3.57e-19; TLR9: r = 0.09, *p* = 0.039).

**FIGURE 5:**
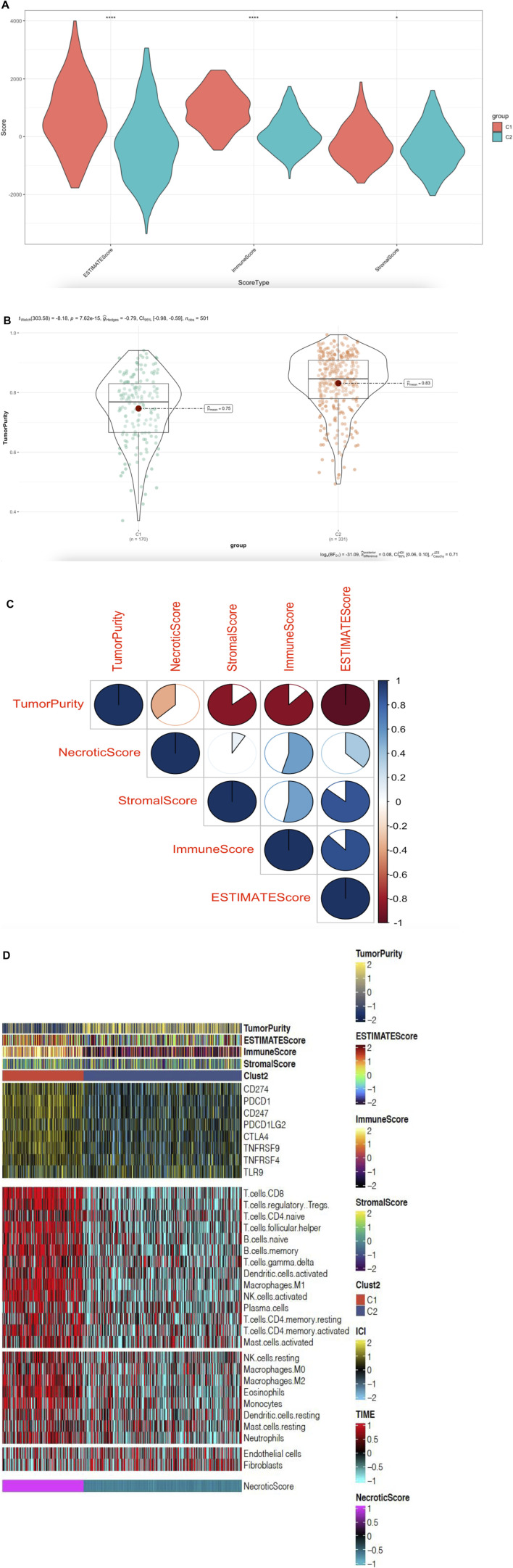
NecroticScore influences the tumor immune microenvironment in HNSCC. **(A)** ESTIMATEScore, ImmuneScore, and StromalScore in C1 and C2 groups; from TCGA; the asterisk represents the different *p* values (* < 0.05; ** < 0.01; *** < 0.001, **** < 0.0001), C1 (170cases), C2 (331cases). **(B)** TumorPurity in C1 and C2 groups; from TCGA; *p* = 7.63e-15, C1 (170cases), C2 (331cases). **(C)** The bubble plot displays the correlation between the NecroticScore and four score types of TME. Red bubbles mean a positive correlation, blue means a negative correlation, the color depth and color size indicate the intensity of the correlation. With the bubble color redder, the positive correlation is higher. **(D)** Complex-heatmap showing the profile in the HNSCC-TCGA cohort, with the top panel showing the expression of genes involved in immune checkpoint targets and the bottom panel showing the enrichment level of 24 microenvironment cell types. ESTIMATEScore, ImmuneScore, StromalScore, TumorPurity, and Clust2 were annotated at the top of the heatmap, NecroticScore was annotated at the bottom of the heatmap.

To quantify the proportions of immune cells and tumor cells we used “ESTIMATE” R package to calculate the score of stromal and immune cells in head and neck squamous cell carcinoma samples. We set the score name to ESTIMATEScore, ImmuneScore, StromalScore, and TumorPurity, which were specific values in order to evaluate the correlation coefficient between the two groups. We found ESTIMATEScore, ImmuneScore, and StromalScore was higher in C1 cluster than C2 cluster, however, C1 cluster had lower TumorPurity than C2 (Figures 5A–B). We also found NecroticScore was positively correlated with ESTIMATEScore, ImmuneScore, and StromalScore, but negatively correlated with TumorPurity (Figure 5C). We detected the verification correlation between NecroticScore and immune cell type by CIBERSORT algorithm (Newman et al., 2015); the landscape of immune cell infiltration is similar to GSVA, and we plotted a combined heat map to display the above results (Figure 5D).

### Construction and Verification of the Necroptosis-Related Molecules Risk Prediction Model

**FIGURE 6:**
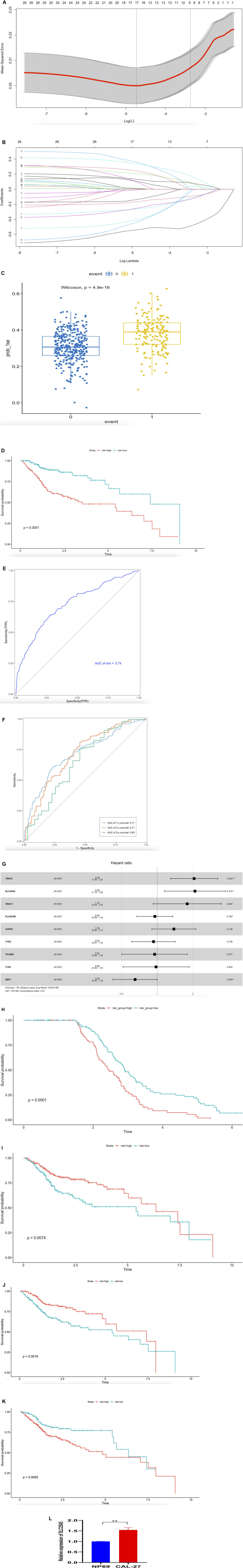
Construction and verification of the necroptosis-related molecules risk prediction model. **(A)** 1000-time cross-validation for tuning parameter selection in the LASSO model; TCGA cohort. **(B)** LASSO coefficient profiles of 26 necroptosis-related genes; TCGA cohort. **(C)** Risk score range in two groups (0 equals alive, 1 equals death); TCGA cohort. **(D)** Datasets assigned to high-risk and low-risk groups based on the risk score. Kaplan–Meier curve for the HNSCC-TCGA cohort, high risk (192cases), low risk (192 cases) Unit of Time (years). **(E)** Receiver operating characteristic (ROC) curve (AUC = 0.74); TCGA cohort. **(F)** TIME-ROC curve in HNSCC-TCGA cohort. **(G)** Plot in multivariate Cox regression and some parameters of the nine necroptosis-related genes signature in GSE65858 database (Concordance index = 0.61, log rank *p* = 0.00417). **(H)** HNSCC patients in high-risk and low-risk groups based on the risk score. Kaplan–Meier curve for the GSE65858 cohort, high risk (134cases), low risk (133 cases), Unit of Time (years). **(I)** HNSCC patients in high-risk and low-risk groups based on the expression of TRAF5. Kaplan–Meier curve for the TCGA cohort, high risk (235cases), low risk (149 cases), Unit of Time (years). **(J)** HNSCC patients in high-risk and low-risk groups based on the expression of ZBP1. Kaplan–Meier curve for the TCGA cohort, high risk (177cases), low risk (207 cases), Unit of Time (years). **(K)** HNSCC patients in high-risk and low-risk groups based on the expression of SLC25A5. Kaplan–Meier curve for the TCGA cohort, high risk (234cases), low risk (150 cases), Unit of Time (years). **(L)** SLC25A5 expression in normal nasopharyngeal epithelial cell line (NP69) cell line and HNSCC cell line (CAL-27), quantitative real-time PCR assay, **p < 0.01.

Several studies reported that some necroptosis-related molecules were biomarkers for prognostic (Gong et al., 2019; Lim et al., 2021). In order to select the most likely candidate prognostic necroptosis-related genes, we performed the LASSO algorithm to identify a set of 26 necroptosis-related genes (Figures 6A–B). The lambda-min value equals 17 necroptosis-related genes and lambda-1se value equals 9 necroptosis-related genes. Considering the convenience of testing, we selected nine necroptosis-related genes (TRAF5, SLC25A5, VDAC1, PLA2G4B, CAPN1, TYK2, TICAM2, TLR3, and ZBP1) to construct a prediction risk model. We used “Predict” function to calculate risk score; the boxplot showed risk score range in two groups (0 equals alive, 1 equals death), we found risk score was higher in group death than group alive (Figure 6C). Then we divided HNSCC-TCGA cohort patients into two score groups named high-risk and low-risk according to the median of risk score; the Kaplan–Meier analysis results showed that the high-score group had significantly higher mortality than the low-score group (Figure 6D). Receiver operating characteristic (ROC) curve analysis was performed to assess the sensitivity and specificity of this risk prediction model (Figure 6E). We calculated the AUC result to validate the precision of the established risk prediction model (Figure 6F) (1 year AUC = 0.71, 3 years AUC = 0.71, 5 years AUC = 0.65). Moreover, the validation cohort was performed by GSE65858; we used multivariate Cox regression analyses to test the above nine necroptosis-related genes. The results reflected TRAF5, SLC25A5, and ZBP1 in patients with HNSCC cancer were associated with survival and could be an important predictor for overall survival (*p* = 0.002, HR = 2.05; *p* = 0.016, HR = 2.08; *p* = 0.049, HR = 0.65; Figure 6G). We also divided patients into high-risk and low-risk score groups according to the median risk score; the Kaplan–Meier analysis results indicated that nine necroptosis-related genes in patients with HNSCC cancer were associated with poor survival (Figure 6H). To validate TRAF5, SLC25A5, and ZBP1 as important predictors of overall survival in TCGA cohort, we plot risk Kaplan–Meier curve according to their expression. We found higher expression of TRAF5 and ZBP1 was associated with good survival (Figures 6I–J), However, higher expression in SLC25A5 predicted bad survival (Figure 6K); in addition, the quantitative real-time PCR assay showed SLC25A5 was up regulated in HNSCC cell line (Figure 6L), indicating this could be a carcinogenesis gene in necroptosis-related molecules.

## Discussion

An increasing number of studies have validated the crucial role of necroptosis in tumor immunity, but the whole mechanism of necroptosis genes in HNSCC is still unclear. In our study, we collected necroptosis-related molecules, analyzed data from TCGA and GSE65858 of head and neck squamous cell carcinoma, and defined two types of necroptosis in HNSCC; our results showed that there were effective differences in the survival rates and immune cell infiltration level in this clusters. Previous research (Aaes et al., 2016) showed that cross-priming and proliferation of CD8+ T cells will trigger necroptotic tumor cells enhanced antitumor immunogenicity. In addition, authors believed that necroptotic tumor cells serve as potent immunizers in a prophylactic tumor vaccination model, which is an essential step in confirming that the cell death type is immunogenic (Galluzzi et al., 2017). Furthermore, genes involved in type I IFN response were substantially enriched in necroptotic molecules-sufficient tumor cells, whereas necroptotic molecules absence limited the induction of type I IFN response–relevant genes in tumor cells (Yang Y. et al., 2021), and type I IFNs have been certified to play a critical role in the functions of antitumor and immunity (Demaria et al., 2019). This illustrated that necroptotic molecules will trigger type I IFN responses and remold the tumor microenvironment (TME). We constructed a set of scoring systems named NecroticScore to better classify the expression of necroptosis genes in HNSCC patients and to assess the level of necroptosis. According to the NecroticScore, we divided patients into two groups. Our results revealed that almost all immune cell infiltration levels of the high-NecroticScore group were significantly higher than the low-NecroticScore group. Our study calculated that NecroticScore is reliable for the assessment of HNSCC necroptosis-related genes and the prediction of the prognosis of patients. Our results showed NecroticScore is positively correlated with ESTIMATEScore, ImmuneScore, StromalScore, and, notably, negatively correlated with TumorPurity. These detections indicated a significant correlation between NecroticScore and the tumor immune microenvironment. Our results demonstrated high NecroticScore forecasted high sensitivity to immune checkpoint inhibitors (ICIs) response. In addition, we found NecroticScore was higher in the HPV (+) group than HPV (−) group, combining previous results, that support the conclusion of higher infiltration of B cell in HPV (+) HNSCC and lower infiltration of CD8 T cell in HPV (−) HNSCC (Cillo et al., 2020). Therefore, we believed NecroticScore could classify the immunophenotype of HNSCC, predict the prognosis of HNSCC patients, and promote the medication effects.

Our study also found a meaningful correlation between NecroticScore and immune checkpoint expression. CHUN, N et al. (Chun et al., 2021) found activation of the necroptosis-related molecules synergizes with anti-PD1 administration to destroy checkpoint blockade-resistant murine melanoma in murine melanoma. A clinical trial (Topalian et al., 2012) proved to be more effective in the treatment of anti-PD1 antibodies, which were strongly associated with higher expression of PD-L1 and PD-1 checkpoints. In our study, the high NecroticScore group had the higher immune checkpoint molecular expression than low NecroticScore group. Moreover, NecroticScore was positively related with eight immune checkpoint genes and predicted the overall survival in the immunotherapy cohort. Therefore, we speculate a combination of necroptosis and immune checkpoint has great feasibility. We hope our research will contribute to the promotion of new combined therapeutic strategies and serve as the basis for future immunotherapeutic agents.

Given that there is no accuracy of some existing prognostic necroptosis-related signatures in HNSCC cancer, we constructed a predictive model according to necroptosis-related genes and conducted stratification analysis of the overall survival rate for HNSCC patients based on the risk score obtained from the “Predict” function formula in R software. We found that the *p*‐value in the two groups was statistically significant. The validation results showed accuracy and sensitivity of the risk model. So, we do believe this model based on reliable data algorithm will facilitate clinical diagnosis and promote therapy method in HNSCC patients. In addition, we found TRAF5, SLC25A5. and ZBP1 could be important predictors of OS in HNSCC cancer. JIAO, H et al. (Jiao et al., 2020) found ZBP1 could trigger RIPK3-dependent necroptosis and inflammation, which could underlie the development of chronic inflammation. TRAF5 and SLC25A5 were molecules that participate in the signaling cascade downstream of TNFα leading to necroptosis, termed ESCRT complex (Nikoletopoulou et al., 2013). A combination of both the risk Kaplan–Meier curve and quantitative real-time PCR assay indicated that SLC25A5 could play a role in carcinogenesis. However, the specific effects of SLC25A5 in HNSCC cancer have not been reported in research. So, further research is needed.

We recognize some limitations of our research. As collecting fresh clinical sample information for the treatment of patients with HNSCC is difficult, external validations have not been implemented. There is still a lack of research on the specific mechanism of the necroptosis-related molecules involved; further analysis of cellular and molecular assays need to be conducted. But we compensated for this shortcoming through punctilious analysis and using the reliable data algorithm. Moreover, our laboratory is conducting further research on this subject.

## Conclusion

In general, our study suggests that necroptosis plays an important role in tumor microenvironment remodeling, highlighted the associations of the necroptosis-related molecules with changes in the immunological tumor microenvironment in HNSCC, established the normative quantification of the necroptosis genes expression in HNSCC cancer, identified distinct HNSCC cancer immunophenotypes, and may promote HNSCC cancer immunotherapy in the future. We also constructed and validated a necroptosis-related molecules prognostic signature, which proved to have significant value in predicting the overall survival time of HNSCC patients.

## Data Availability

Publicly available datasets were analyzed in this study. This data can be found here: https://portal.gdc.cancer.gov/ and https://www.ncbi.nlm.nih.gov/geo/.
