# Supplementary figures and images for: Necroptosis is Related to Anti-PD-1 Treatment Response and Influences the Tumor Microenvironment in Head and Neck Squamous Cell Carcinoma
Source: Front Genet. 2022 May 25;13:862143. doi: 10.3389/fgene.2022.862143 (PMC9174803; doi:10.3389/fgene.2022.862143)

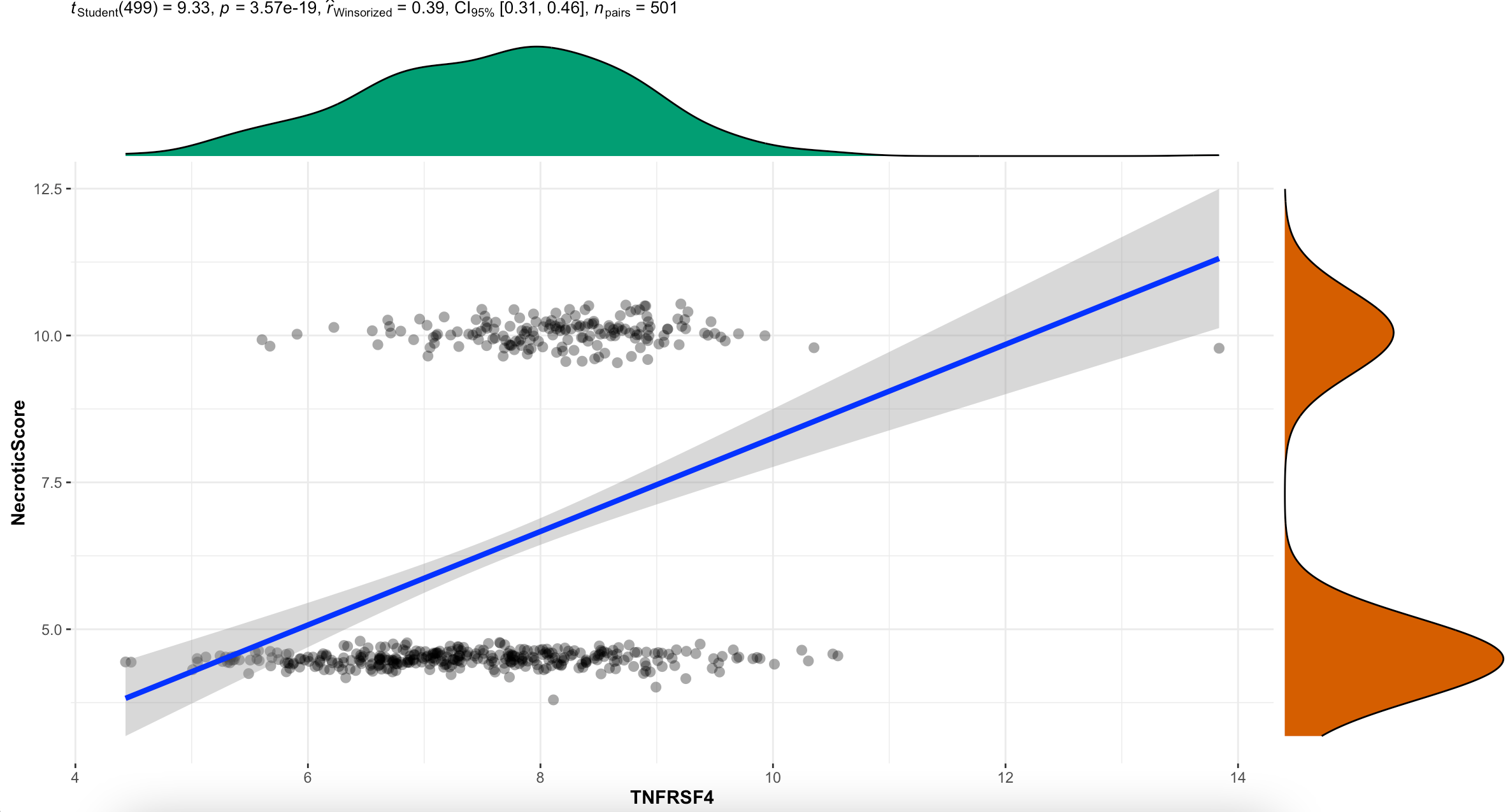

Supplement: Supplementary file 1 [file DataSheet1.ZIP › t c ga/WechatIMG54.png]

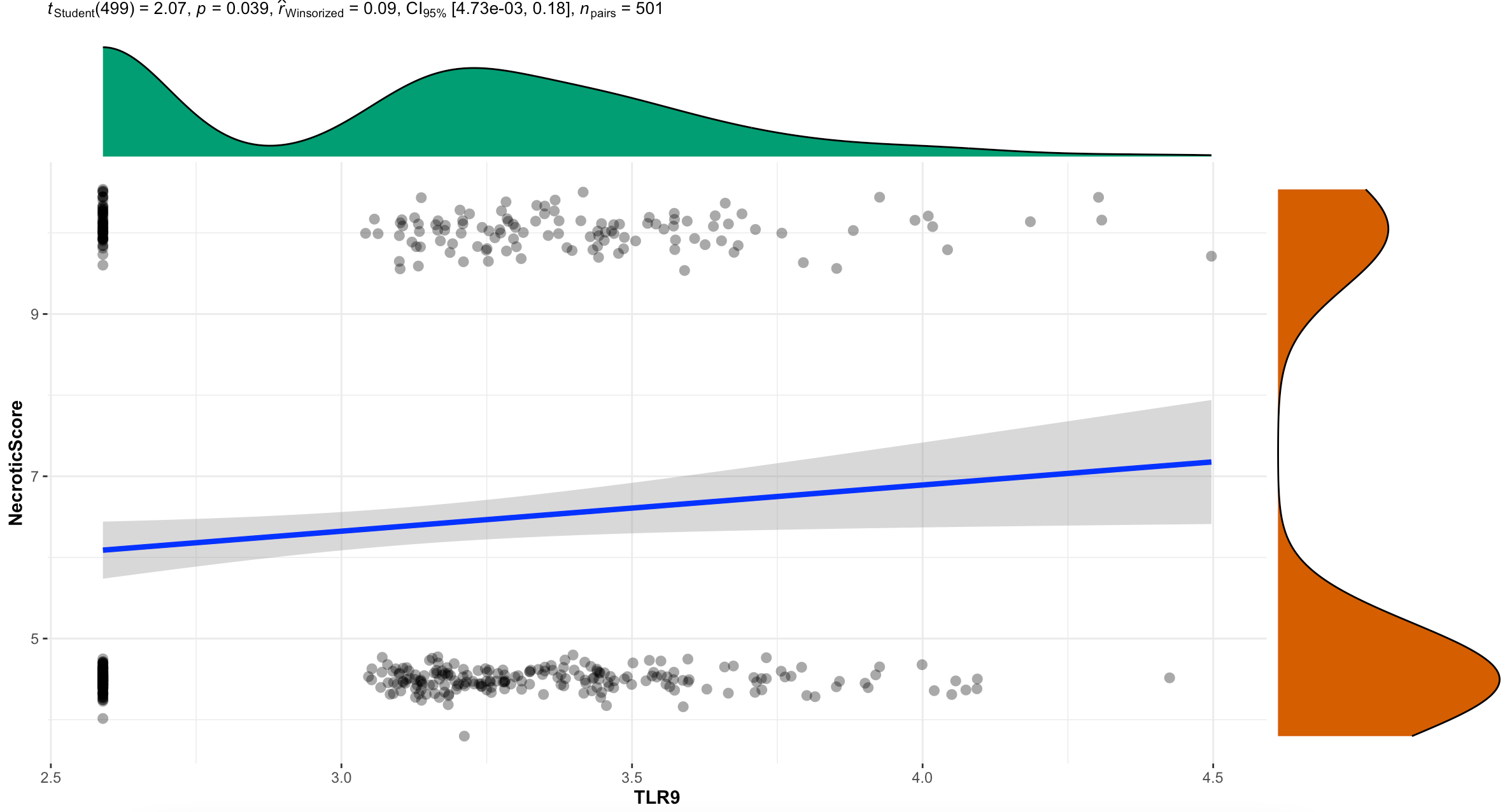

Supplement: Supplementary file 1 [file DataSheet1.ZIP › t c ga/WechatIMG55.png]

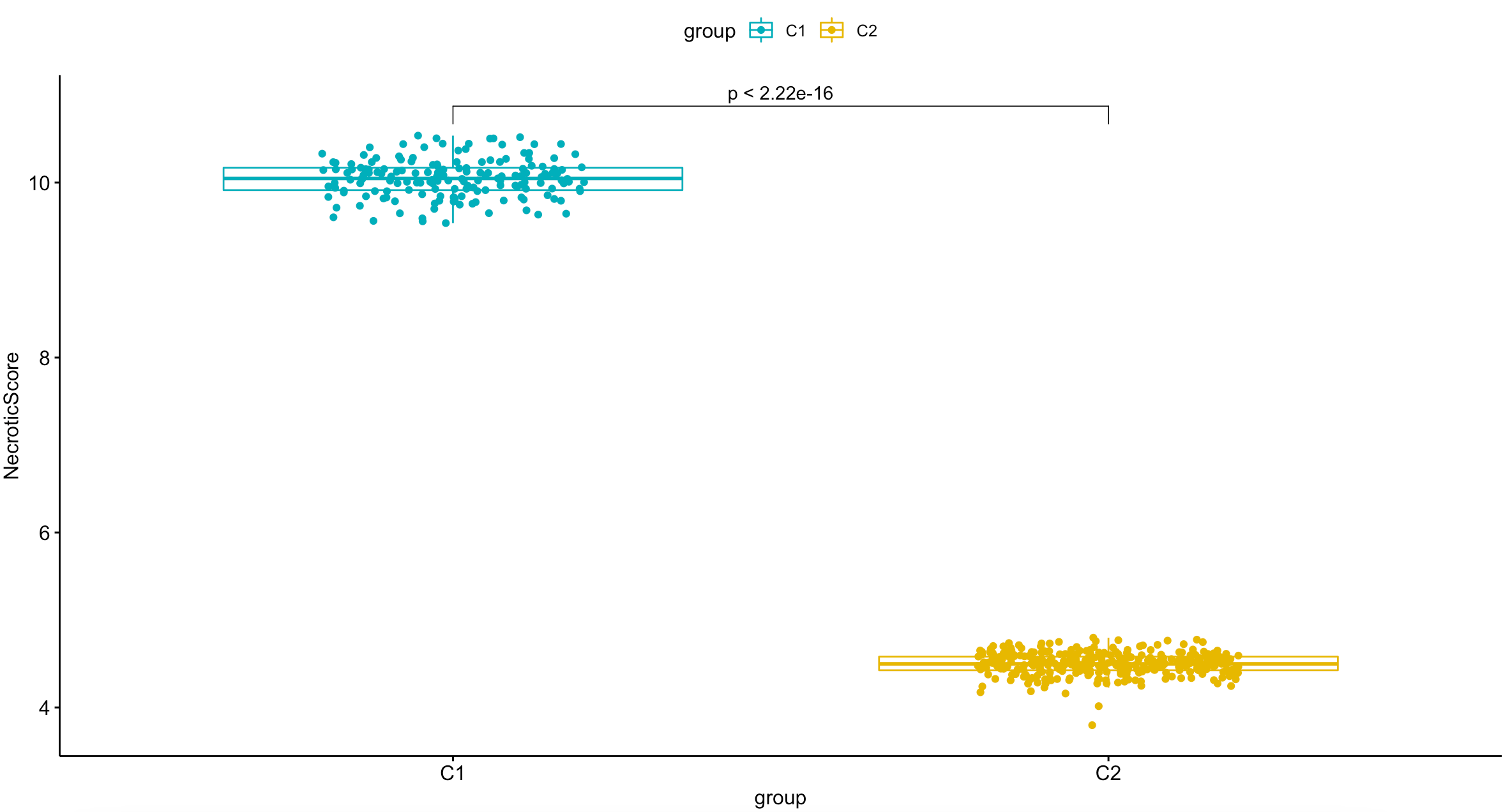

Supplement: Supplementary file 1 [file DataSheet1.ZIP › t c ga/WechatIMG57.png]

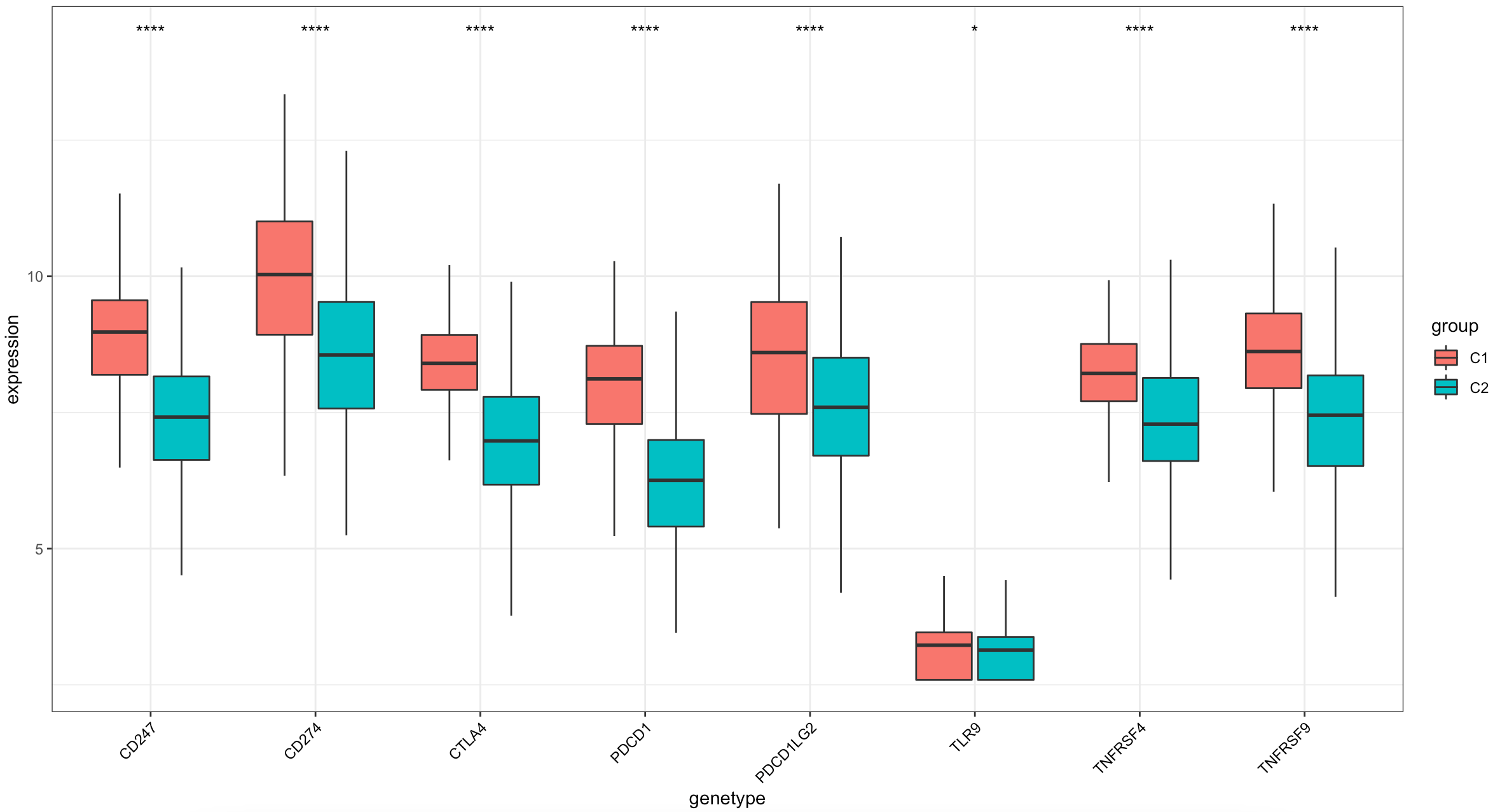

Supplement: Supplementary file 1 [file DataSheet1.ZIP › t c ga/WechatIMG43.png]

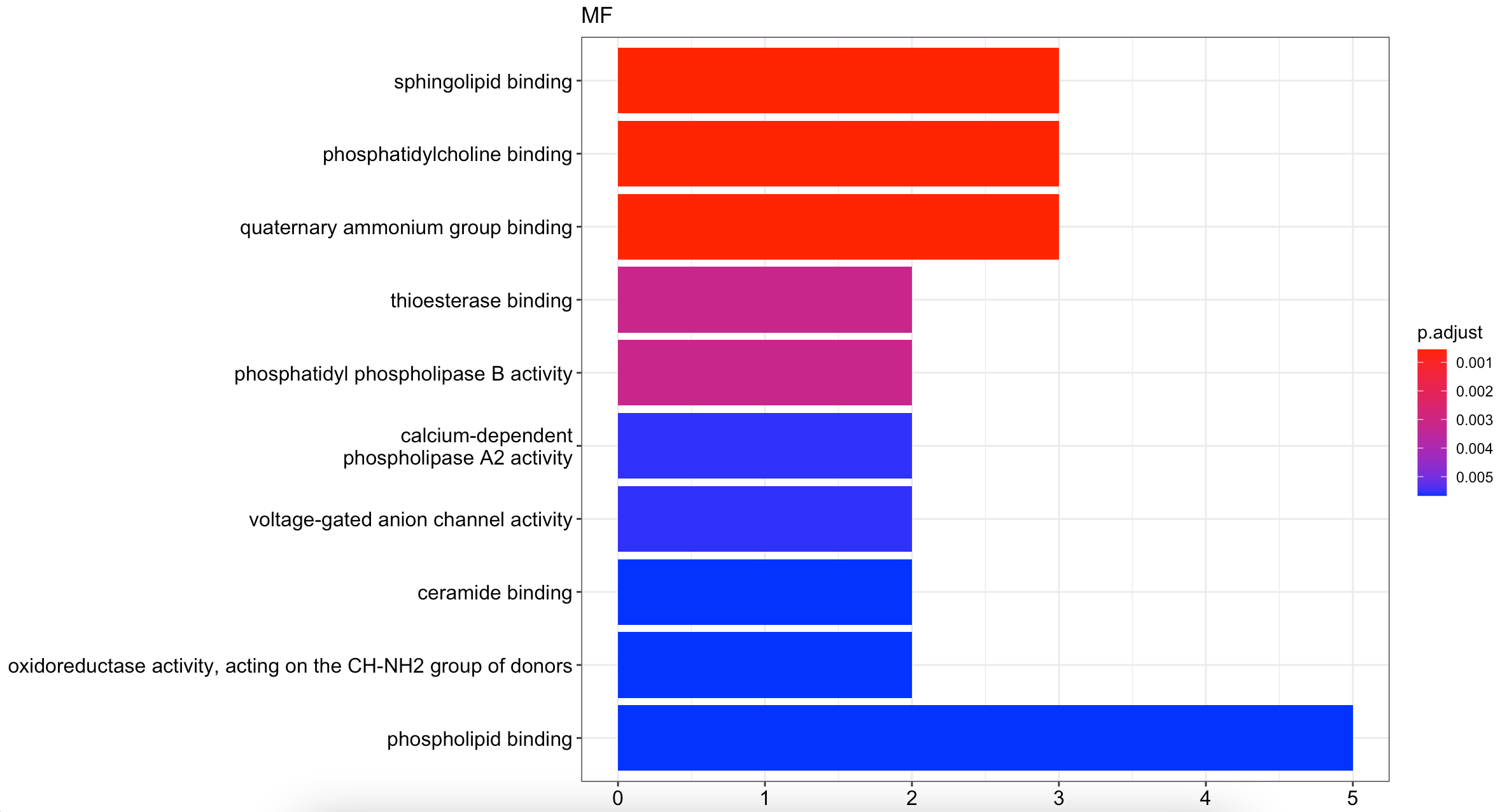

Supplement: Supplementary file 1 [file DataSheet1.ZIP › t c ga/WechatIMG56.png]

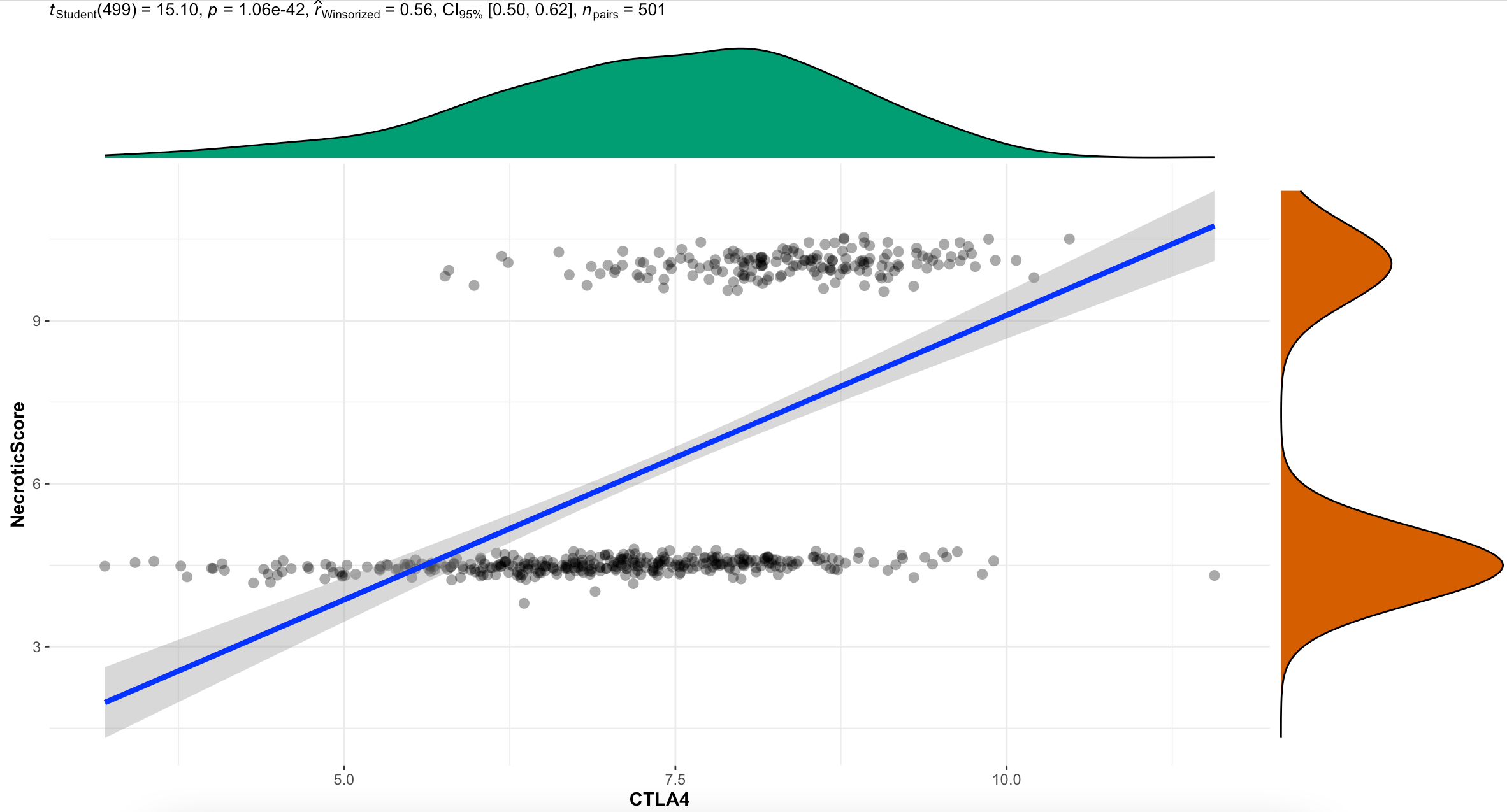

Supplement: Supplementary file 1 [file DataSheet1.ZIP › t c ga/WechatIMG52.png]

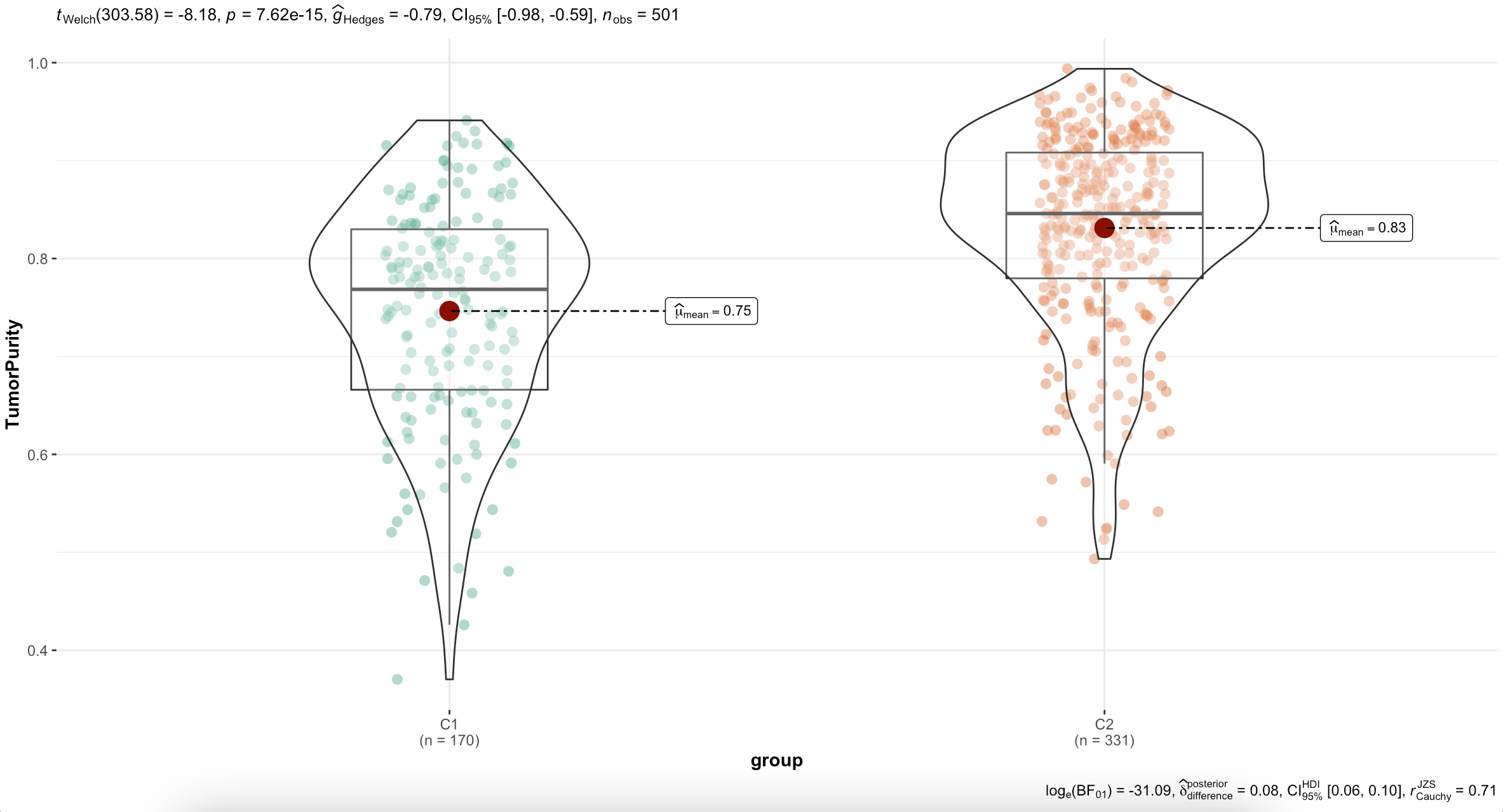

Supplement: Supplementary file 1 [file DataSheet1.ZIP › t c ga/WechatIMG46.png]

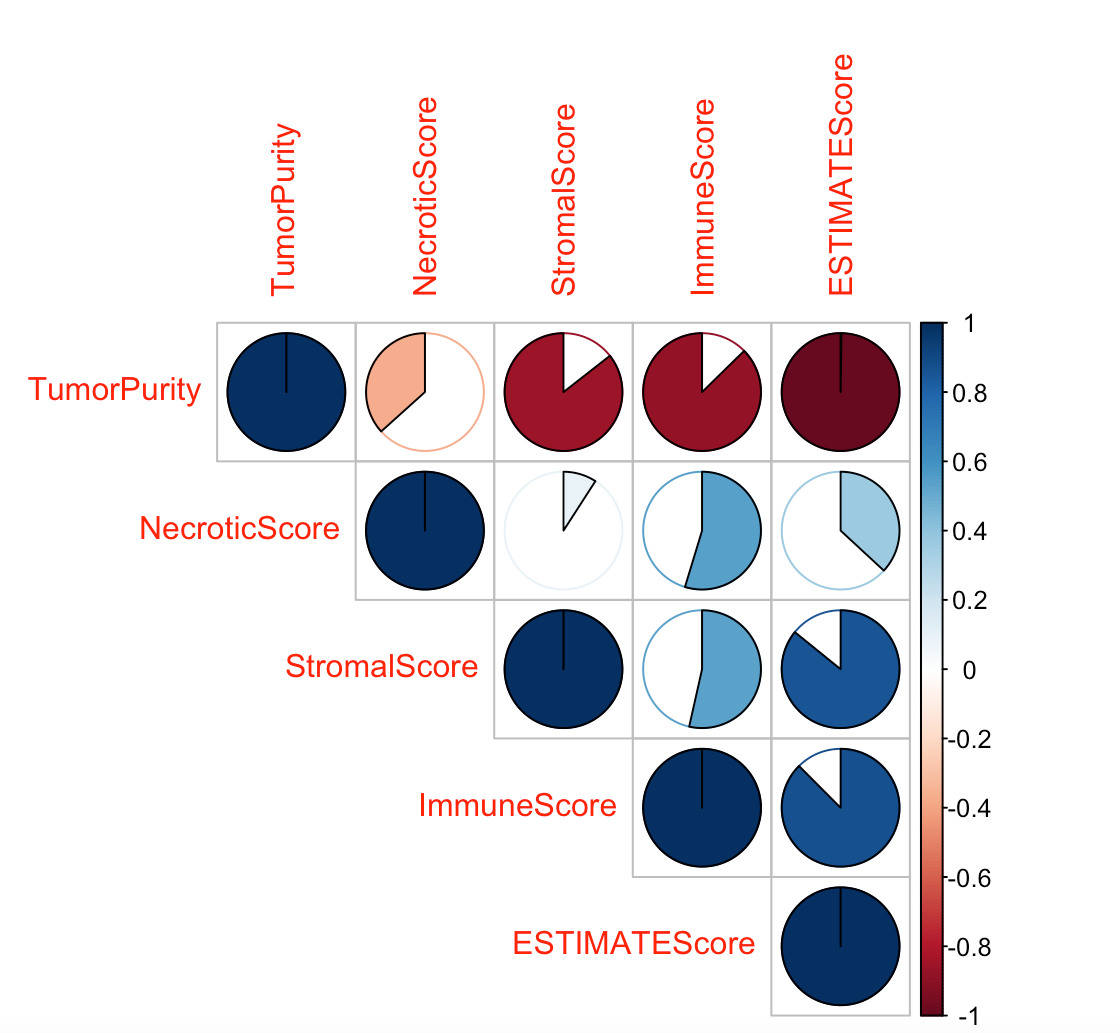

Supplement: Supplementary file 1 [file DataSheet1.ZIP › t c ga/WechatIMG47.png]

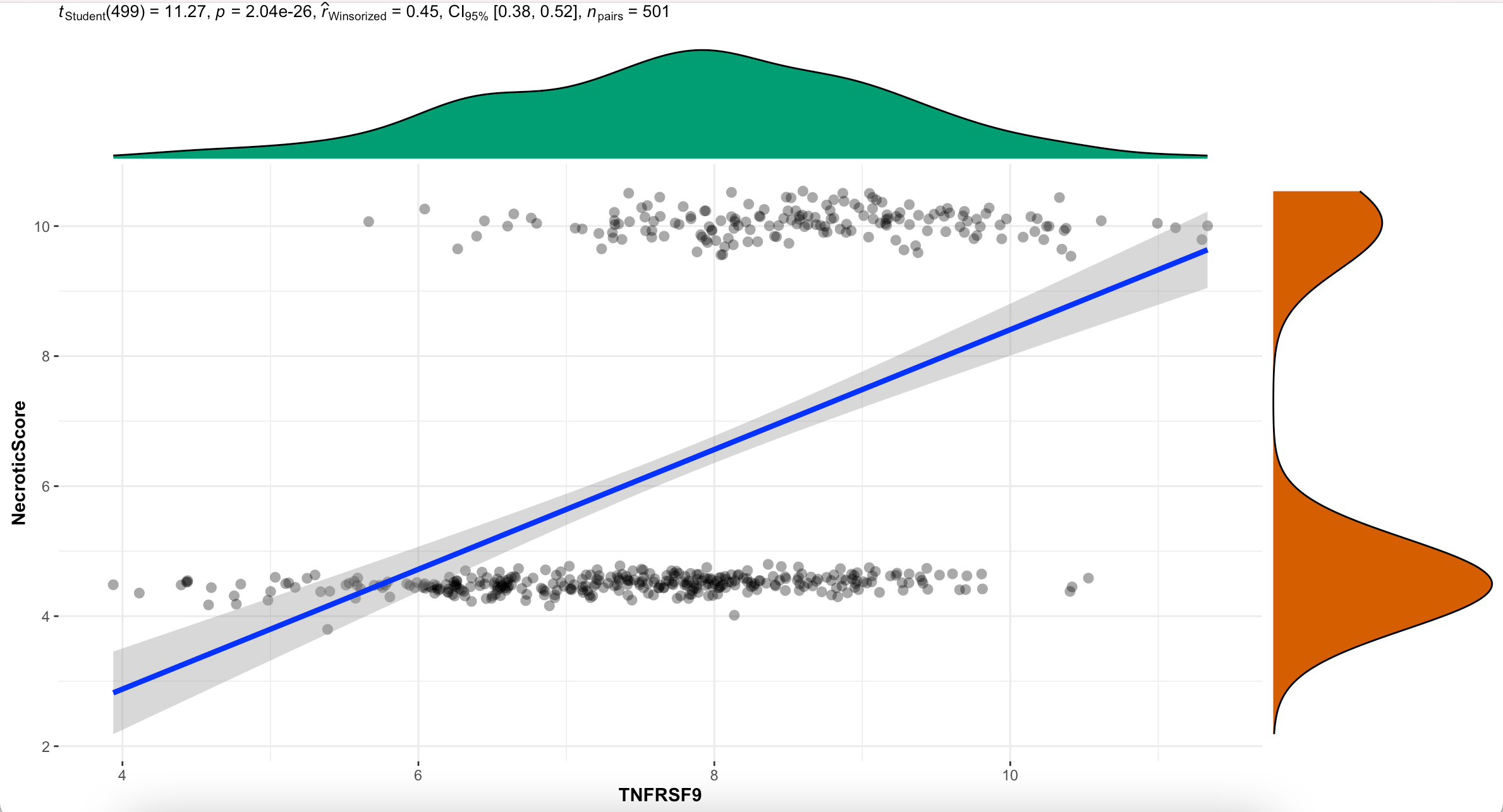

Supplement: Supplementary file 1 [file DataSheet1.ZIP › t c ga/WechatIMG53.png]

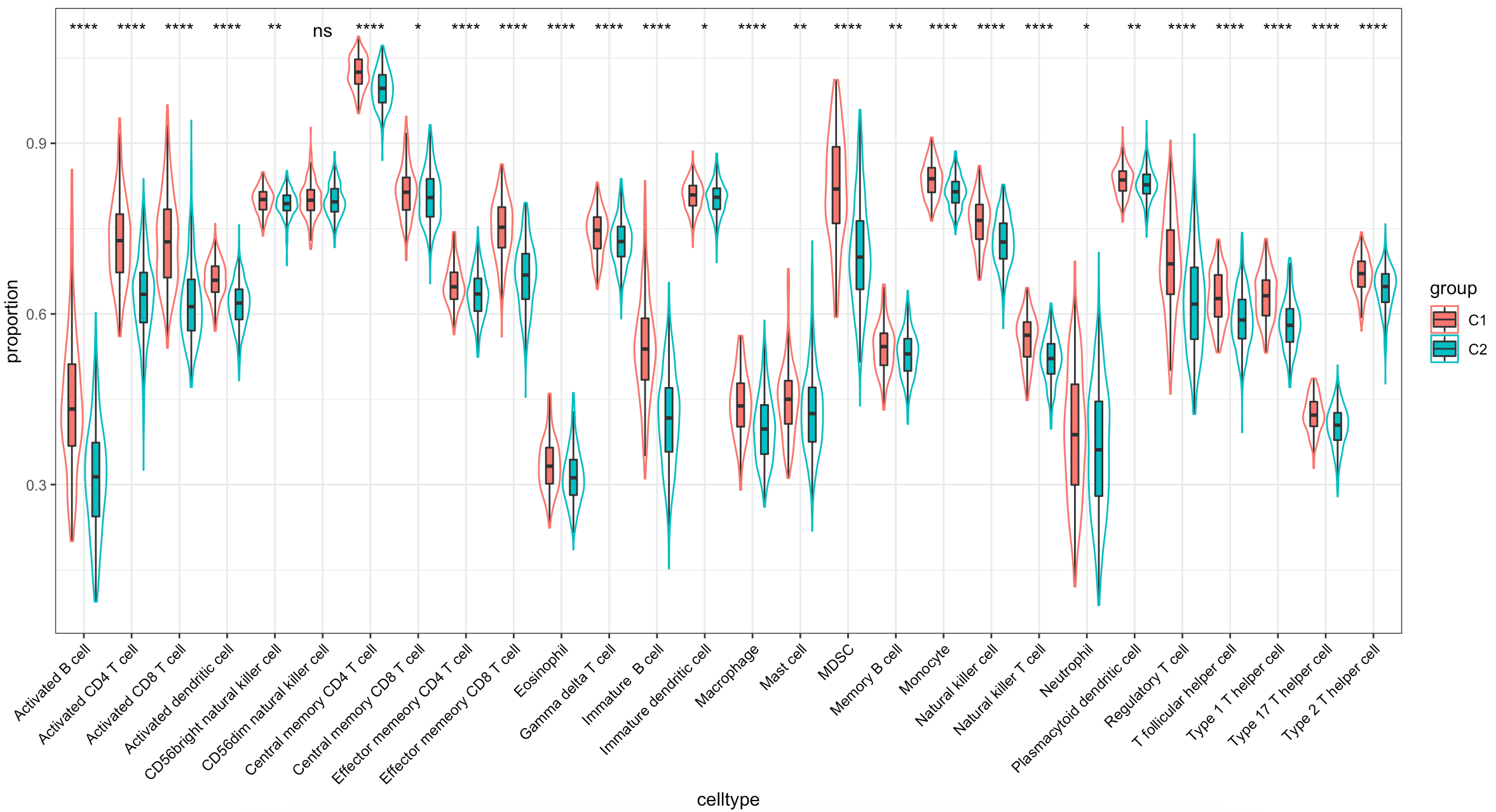

Supplement: Supplementary file 1 [file DataSheet1.ZIP › t c ga/WechatIMG45.png]

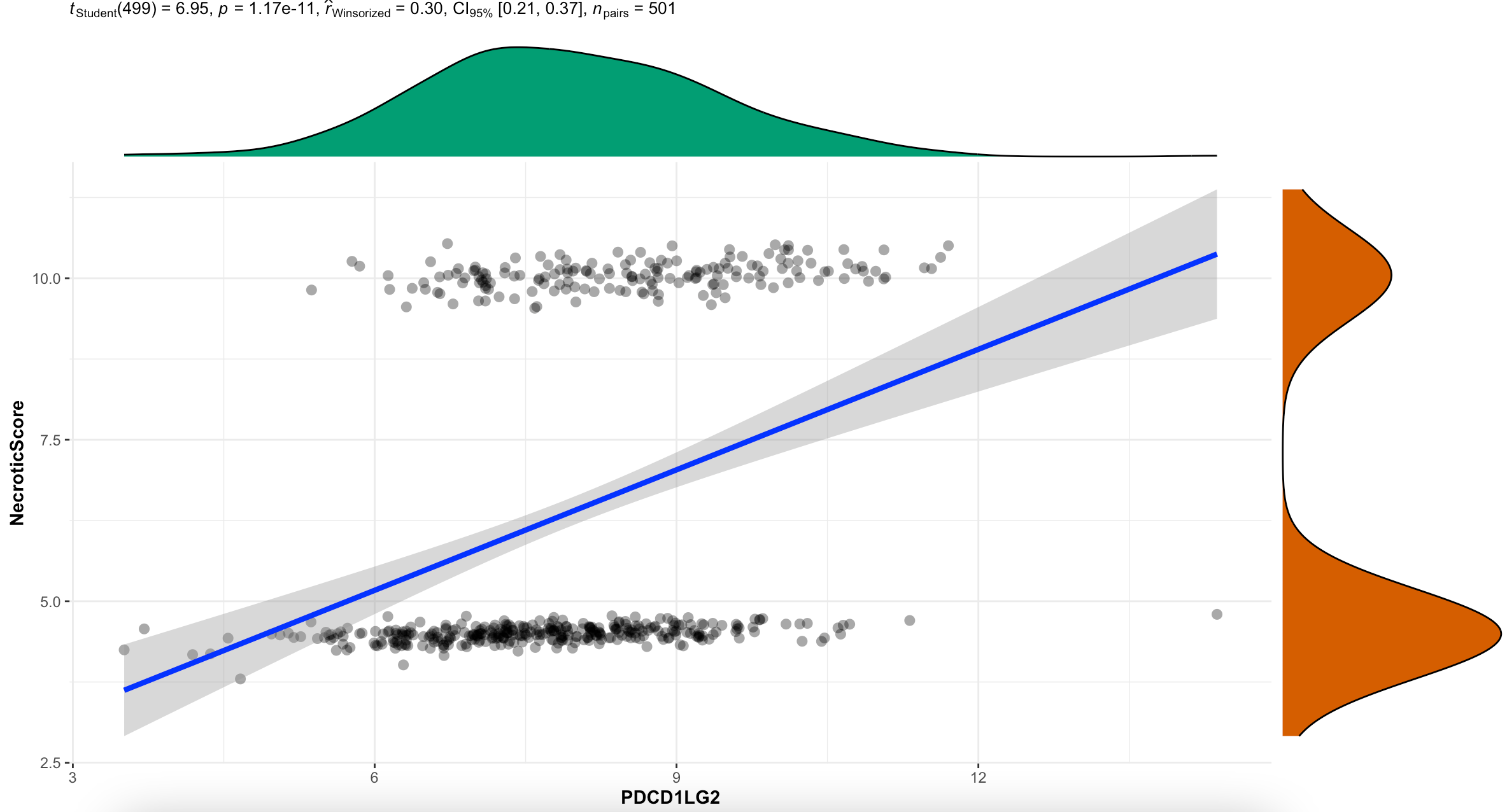

Supplement: Supplementary file 1 [file DataSheet1.ZIP › t c ga/WechatIMG51.png]

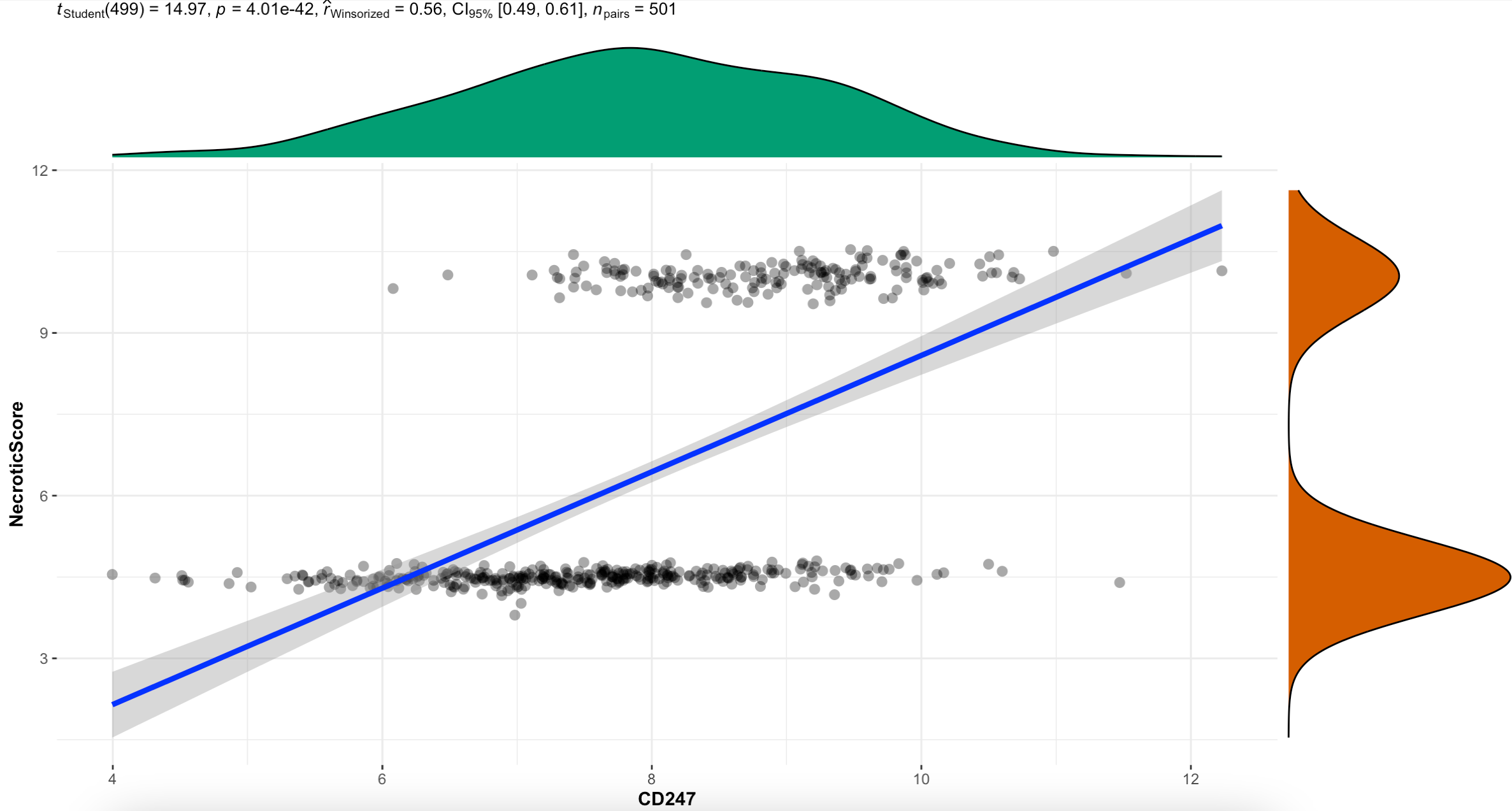

Supplement: Supplementary file 1 [file DataSheet1.ZIP › t c ga/WechatIMG50.png]

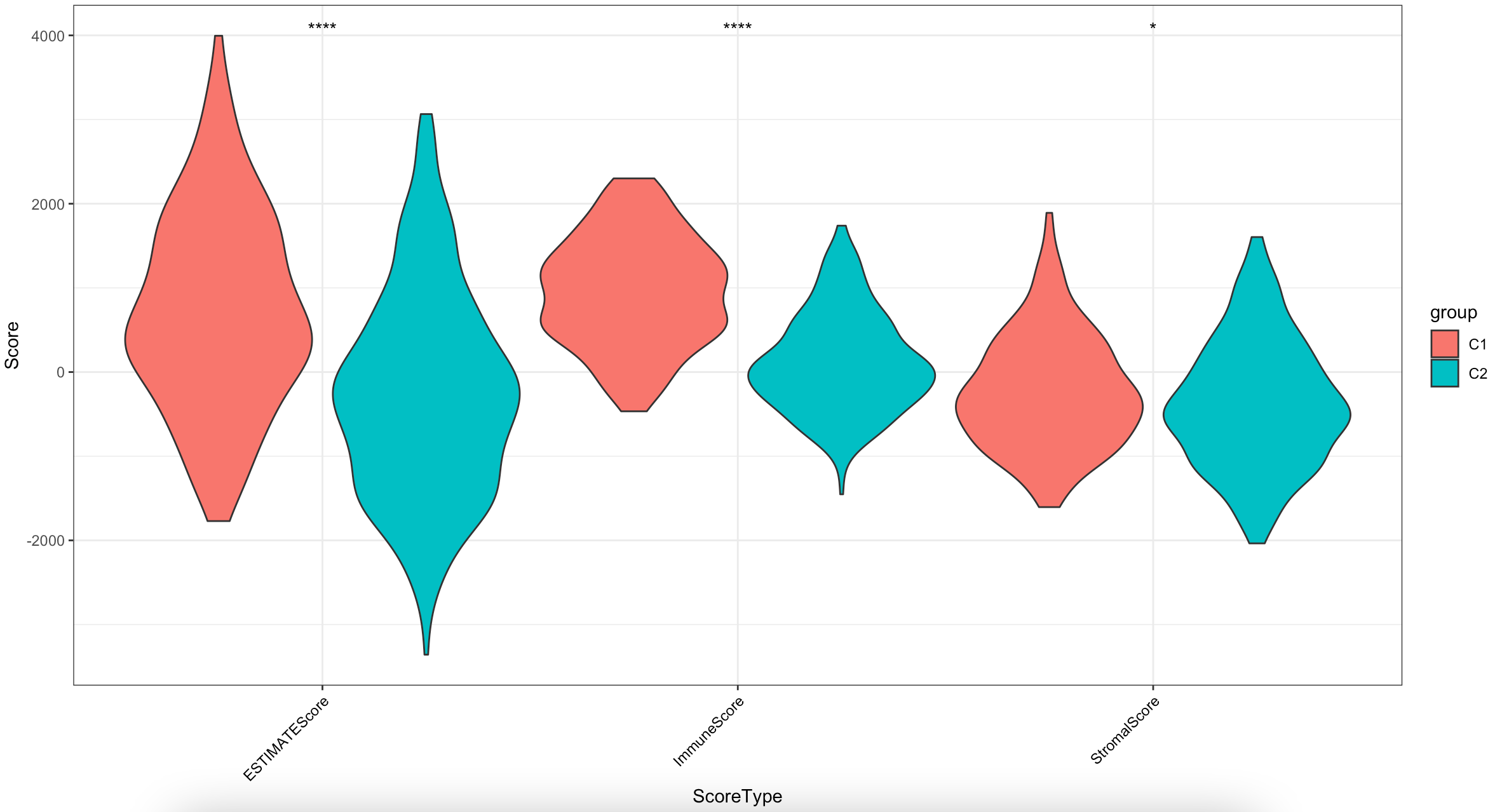

Supplement: Supplementary file 1 [file DataSheet1.ZIP › t c ga/WechatIMG44.png]

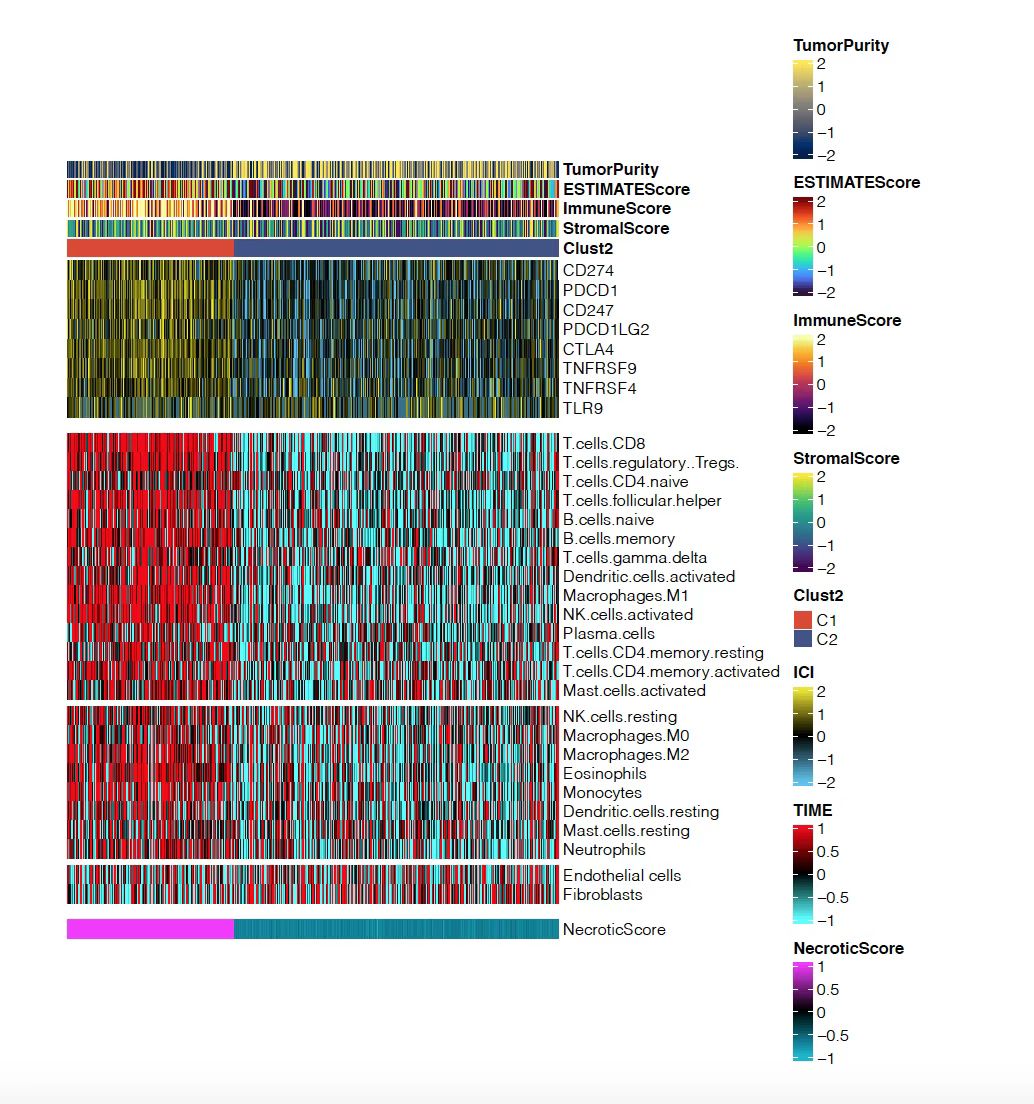

Supplement: Supplementary file 1 [file DataSheet1.ZIP › t c ga/WechatIMG39.jpeg]

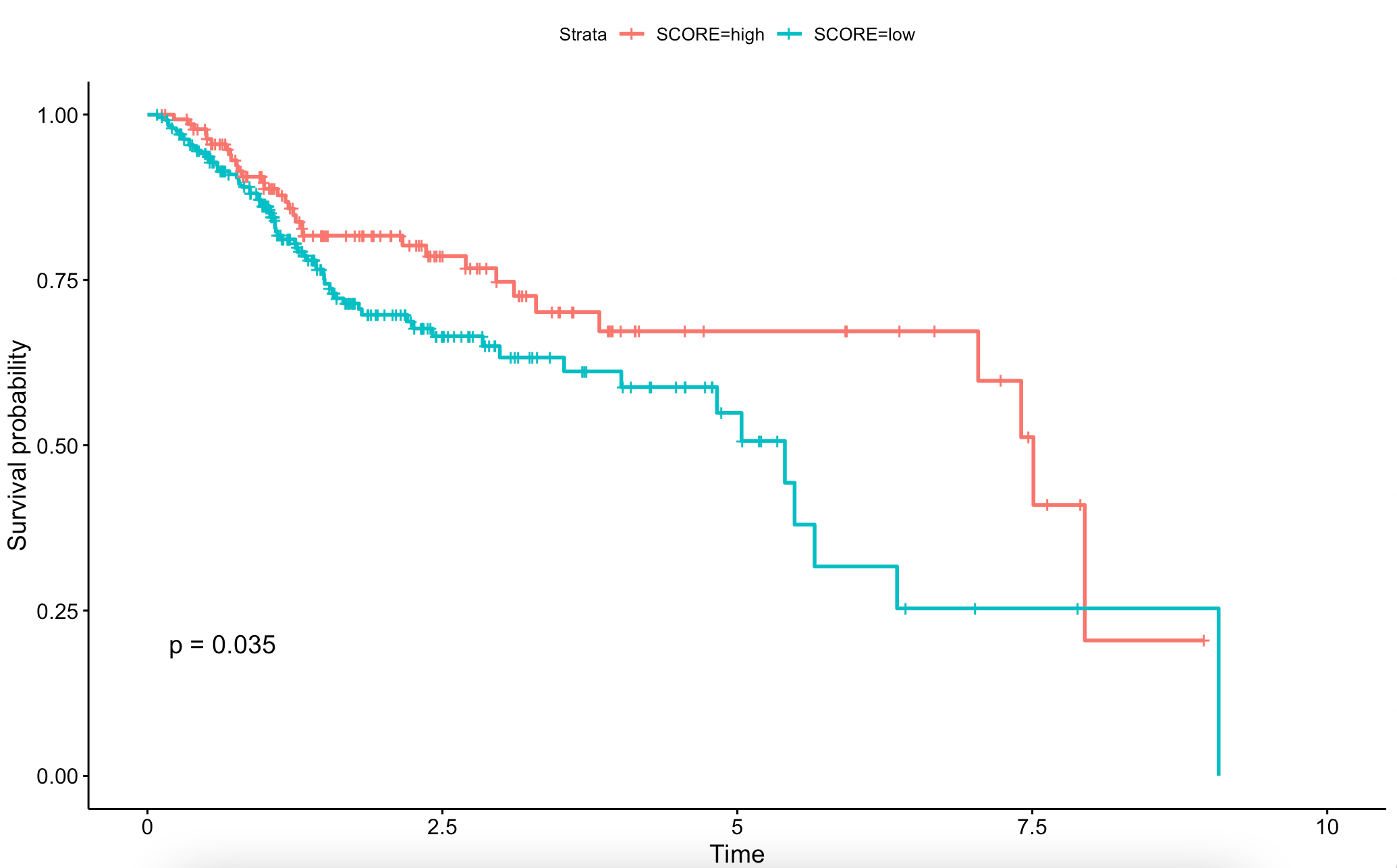

Supplement: Supplementary file 1 [file DataSheet1.ZIP › t c ga/WechatIMG23.png]

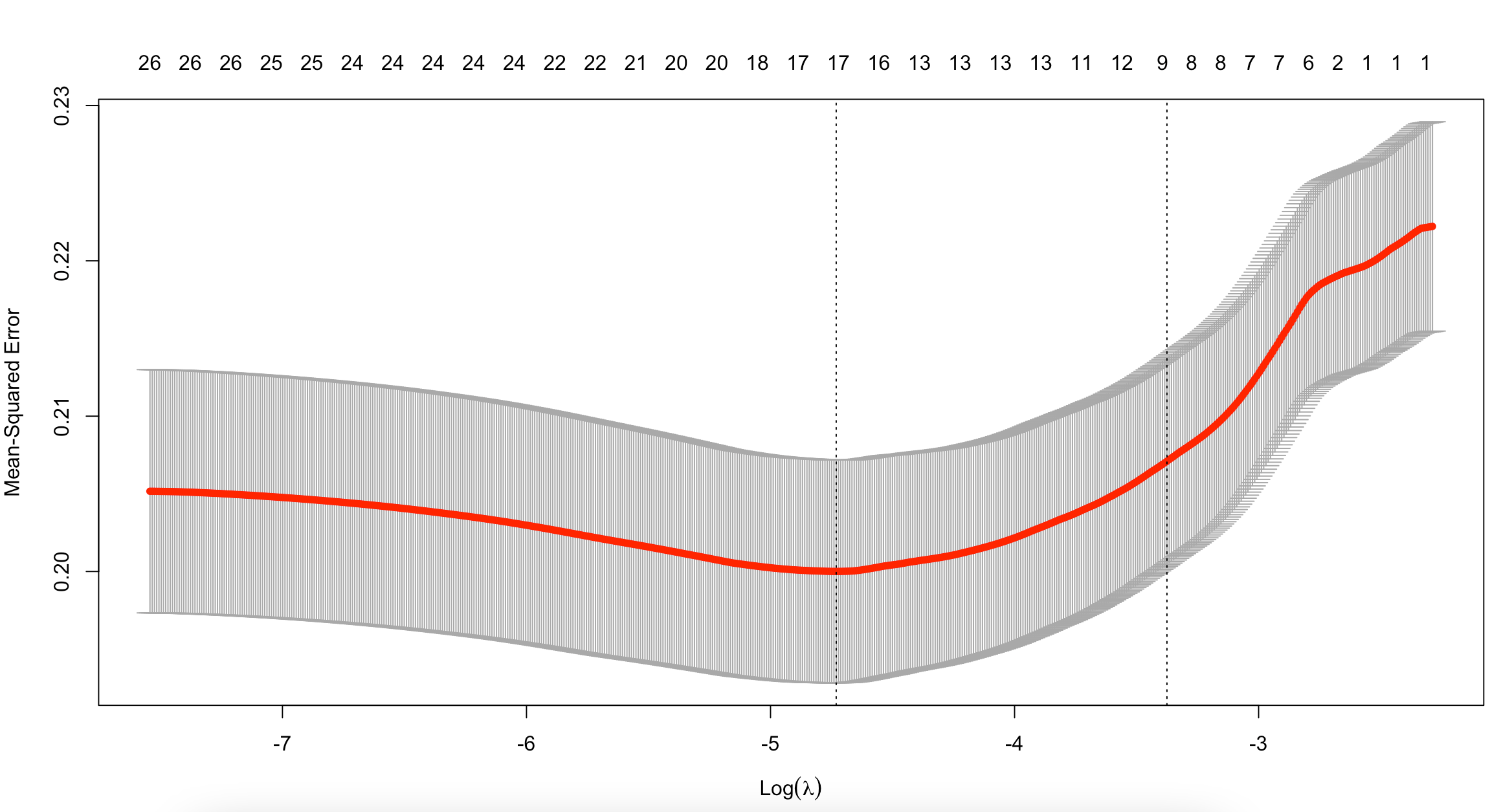

Supplement: Supplementary file 1 [file DataSheet1.ZIP › t c ga/WechatIMG37.png]

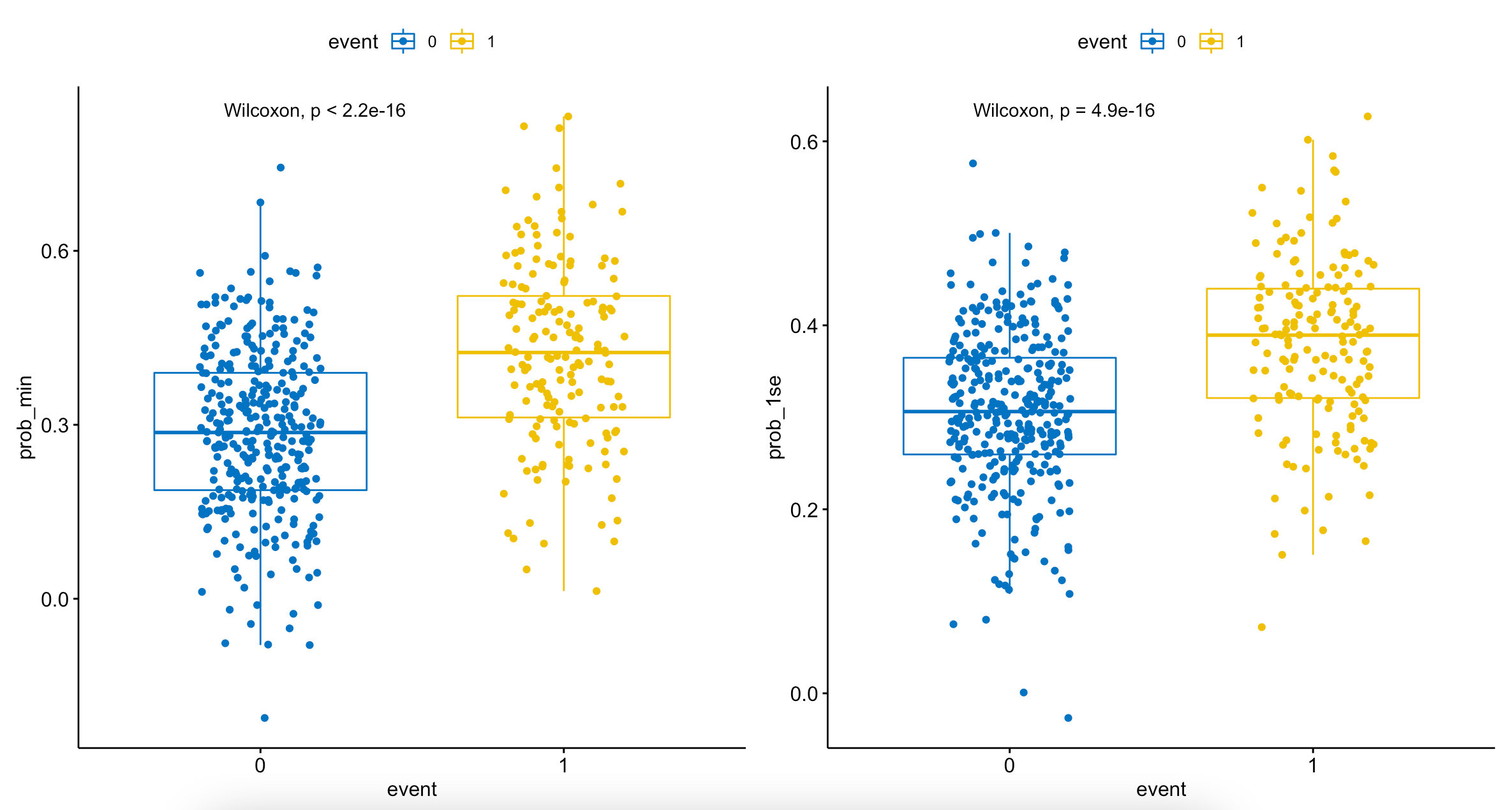

Supplement: Supplementary file 1 [file DataSheet1.ZIP › t c ga/WechatIMG36.png]

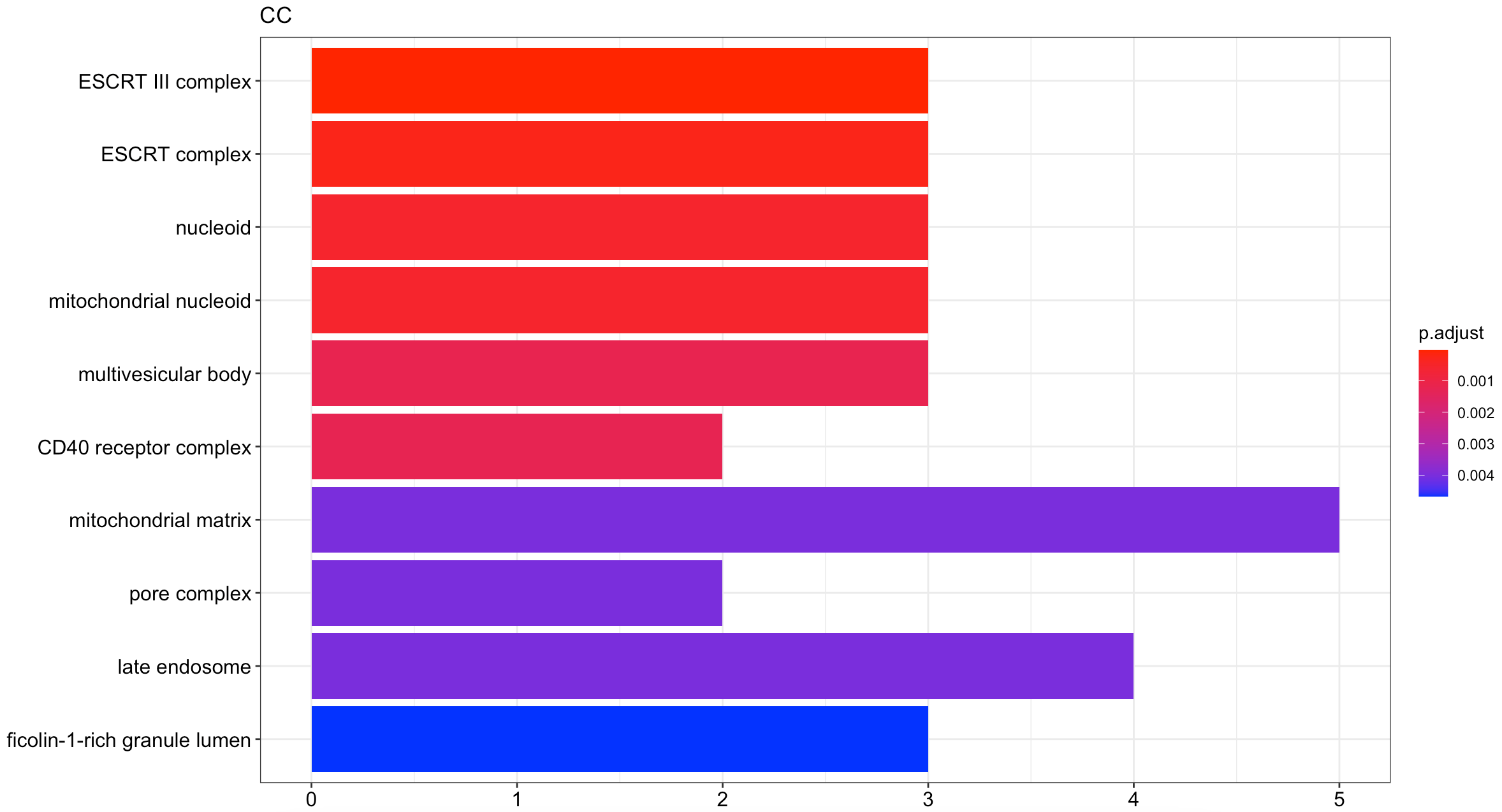

Supplement: Supplementary file 1 [file DataSheet1.ZIP › t c ga/WechatIMG31.png]

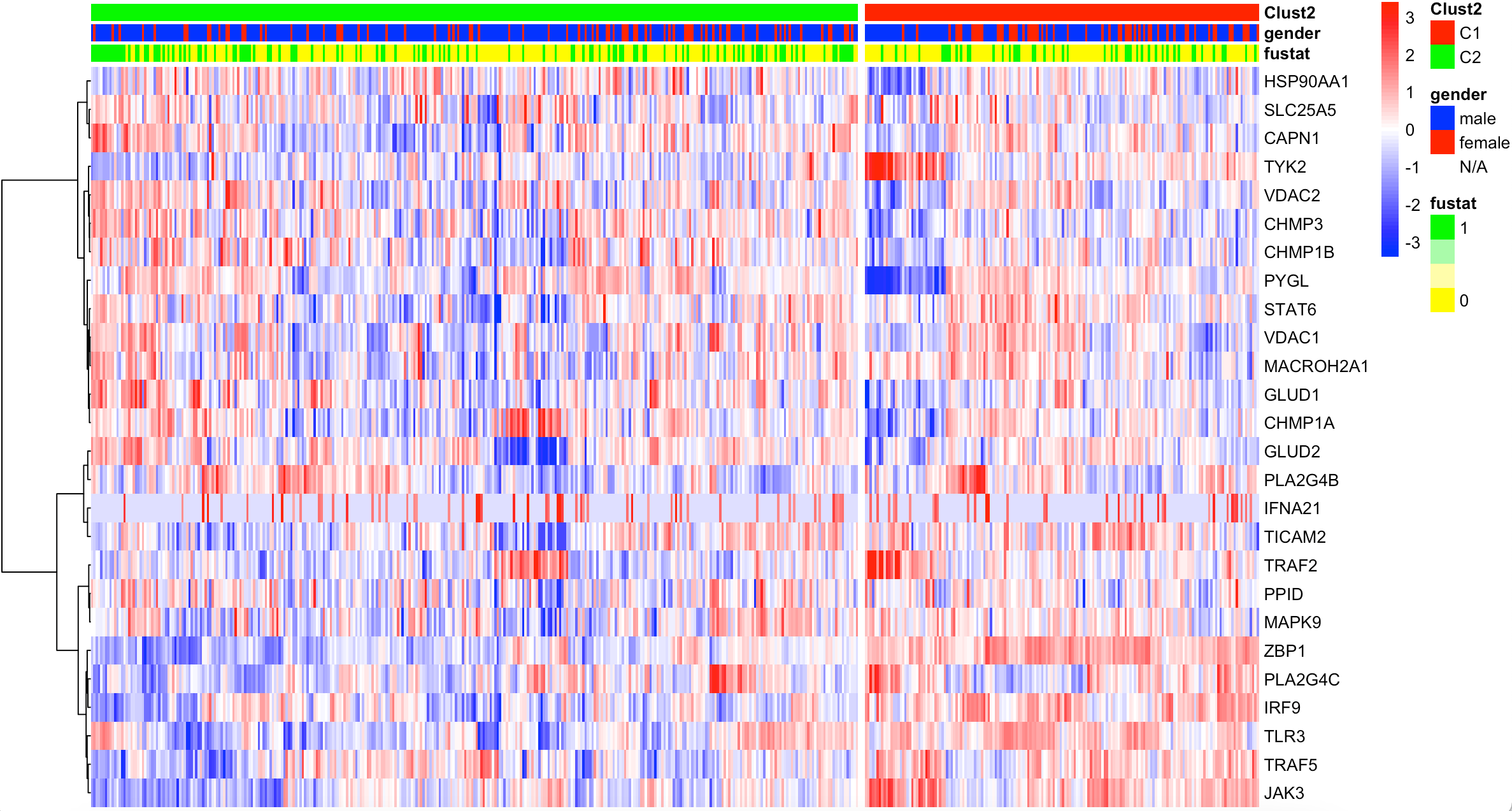

Supplement: Supplementary file 1 [file DataSheet1.ZIP › t c ga/WechatIMG25.png]

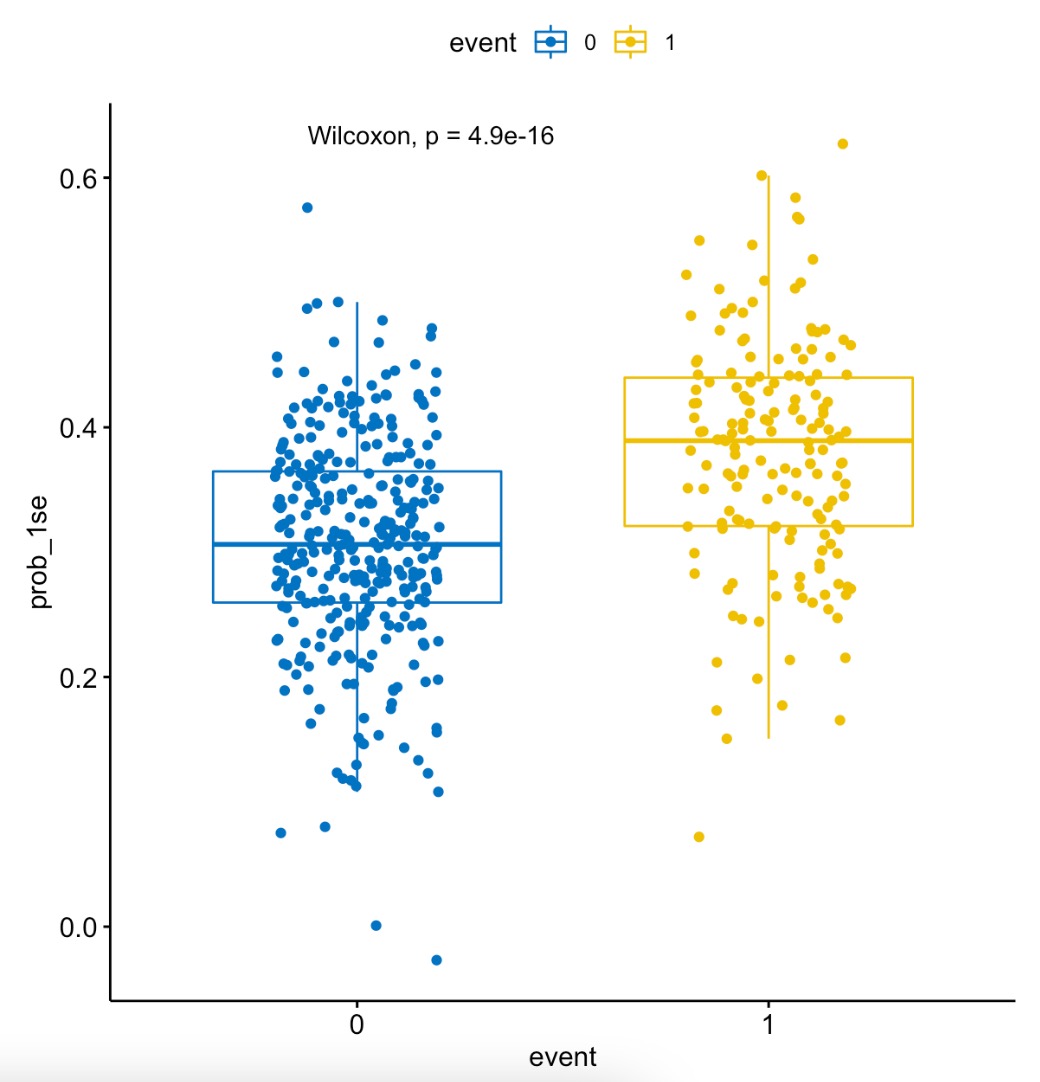

Supplement: Supplementary file 1 [file DataSheet1.ZIP › t c ga/WechatIMG59.jpeg]

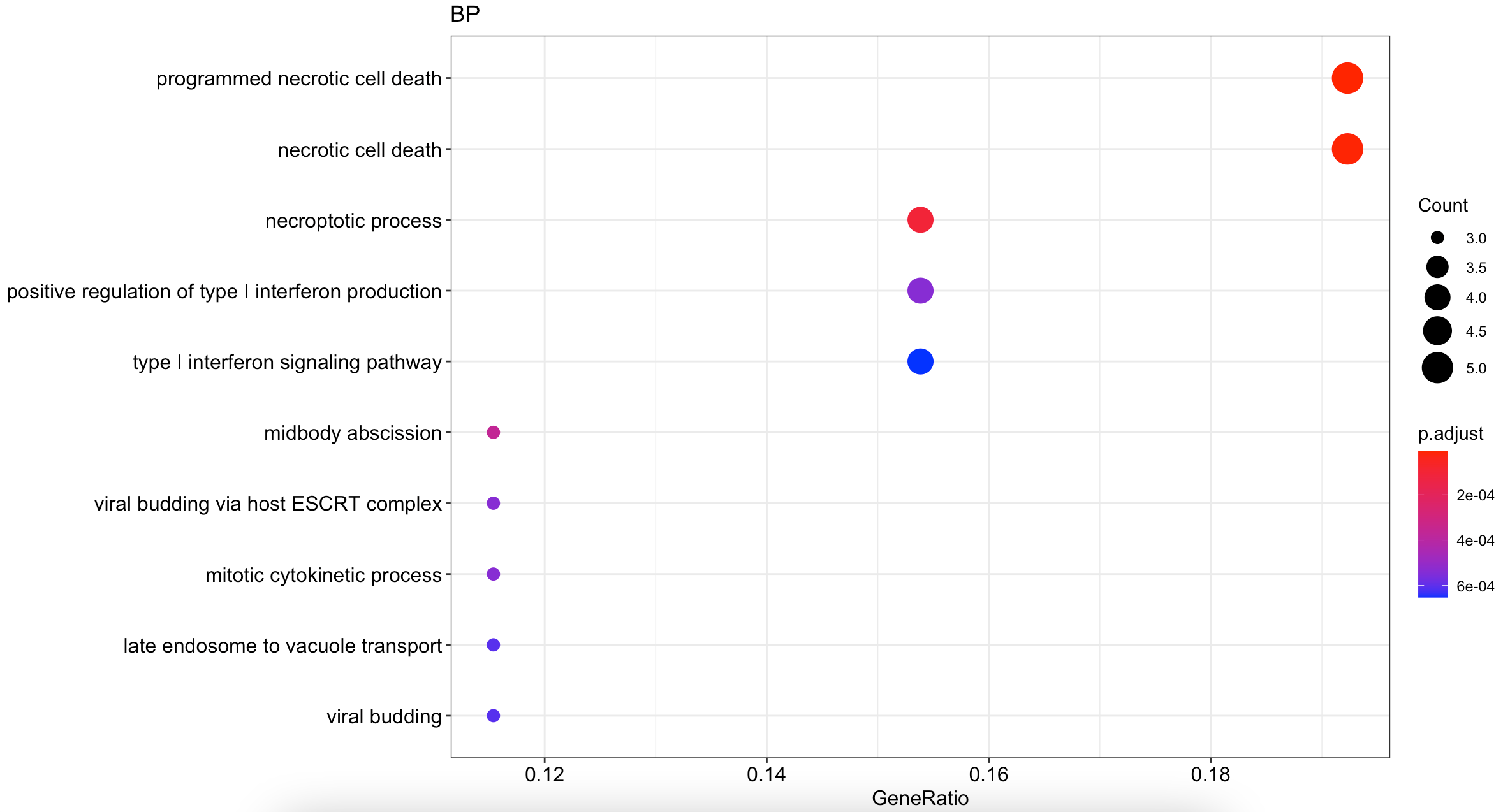

Supplement: Supplementary file 1 [file DataSheet1.ZIP › t c ga/WechatIMG30.png]

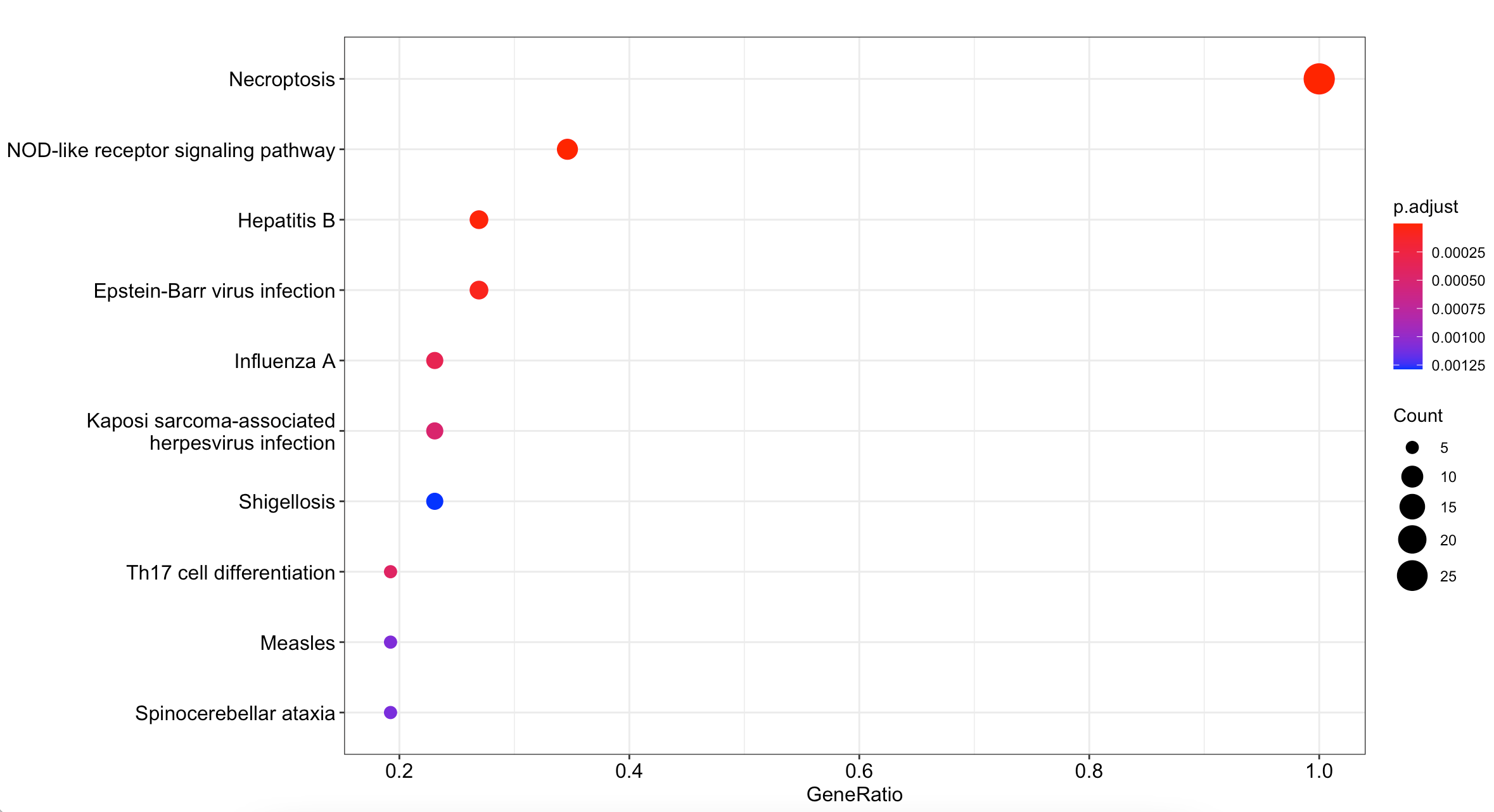

Supplement: Supplementary file 1 [file DataSheet1.ZIP › t c ga/WechatIMG32.png]

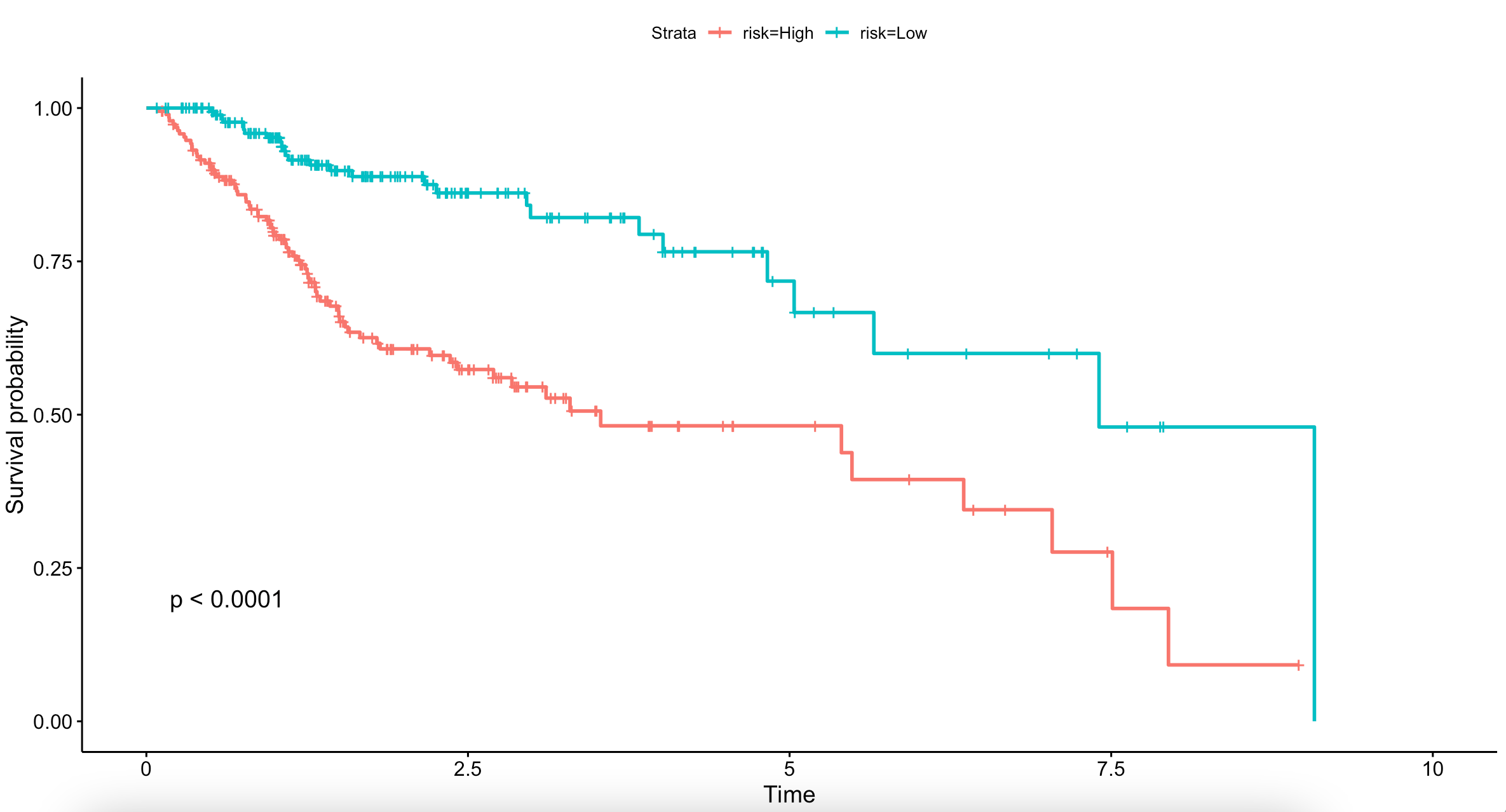

Supplement: Supplementary file 1 [file DataSheet1.ZIP › t c ga/WechatIMG28.png]

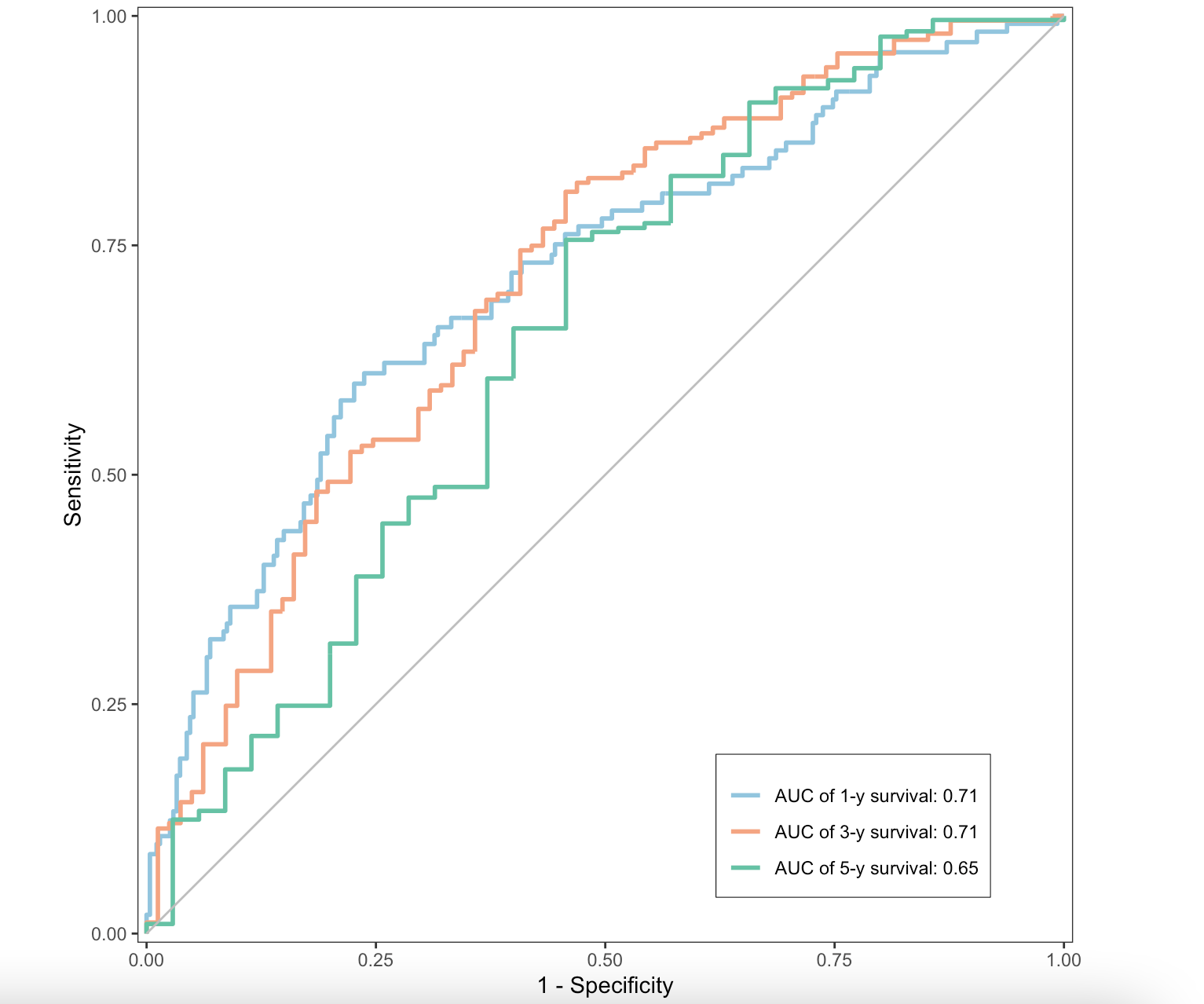

Supplement: Supplementary file 1 [file DataSheet1.ZIP › t c ga/WechatIMG38.png]

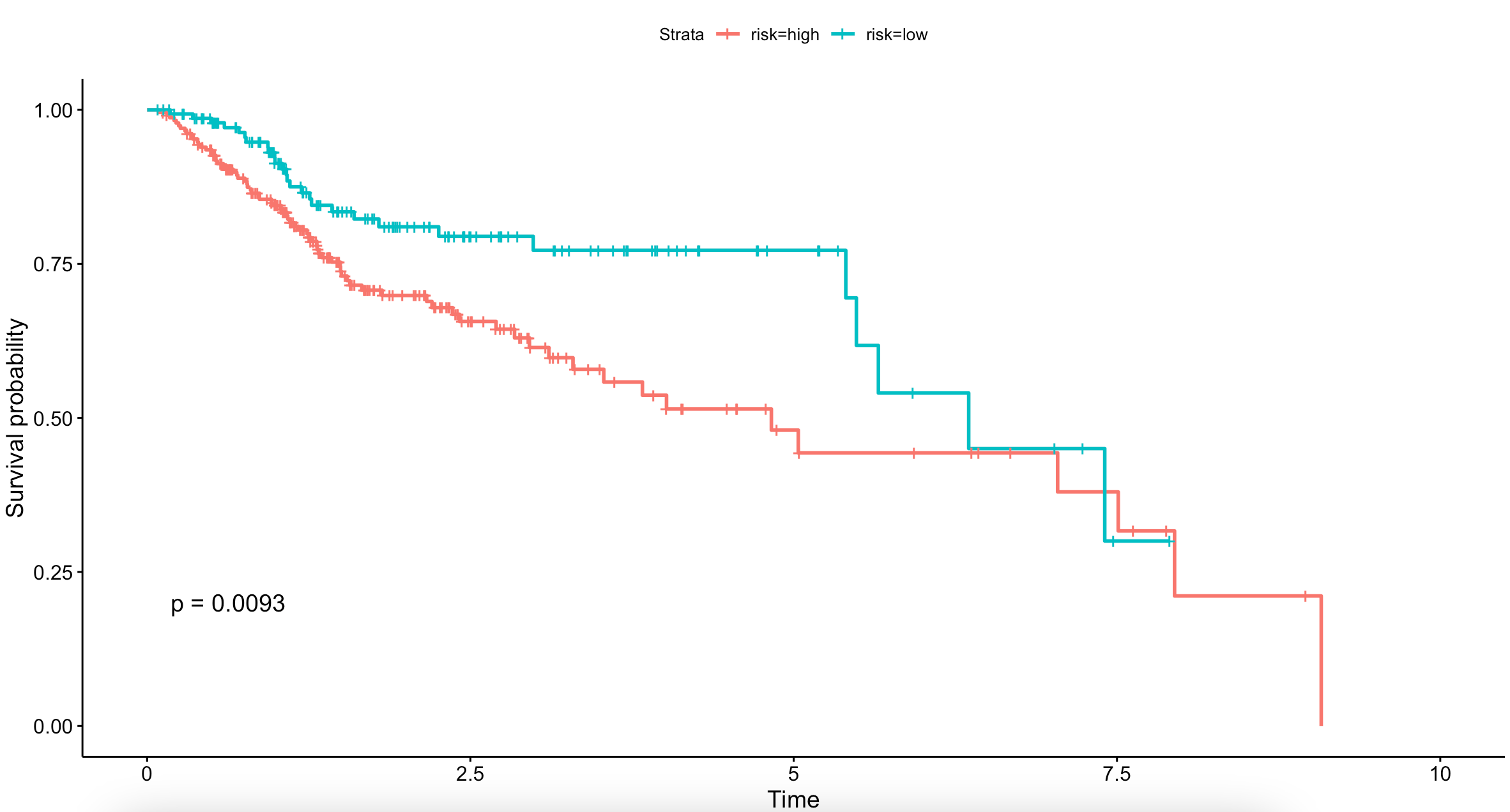

Supplement: Supplementary file 1 [file DataSheet1.ZIP › t c ga/WechatIMG61.png]

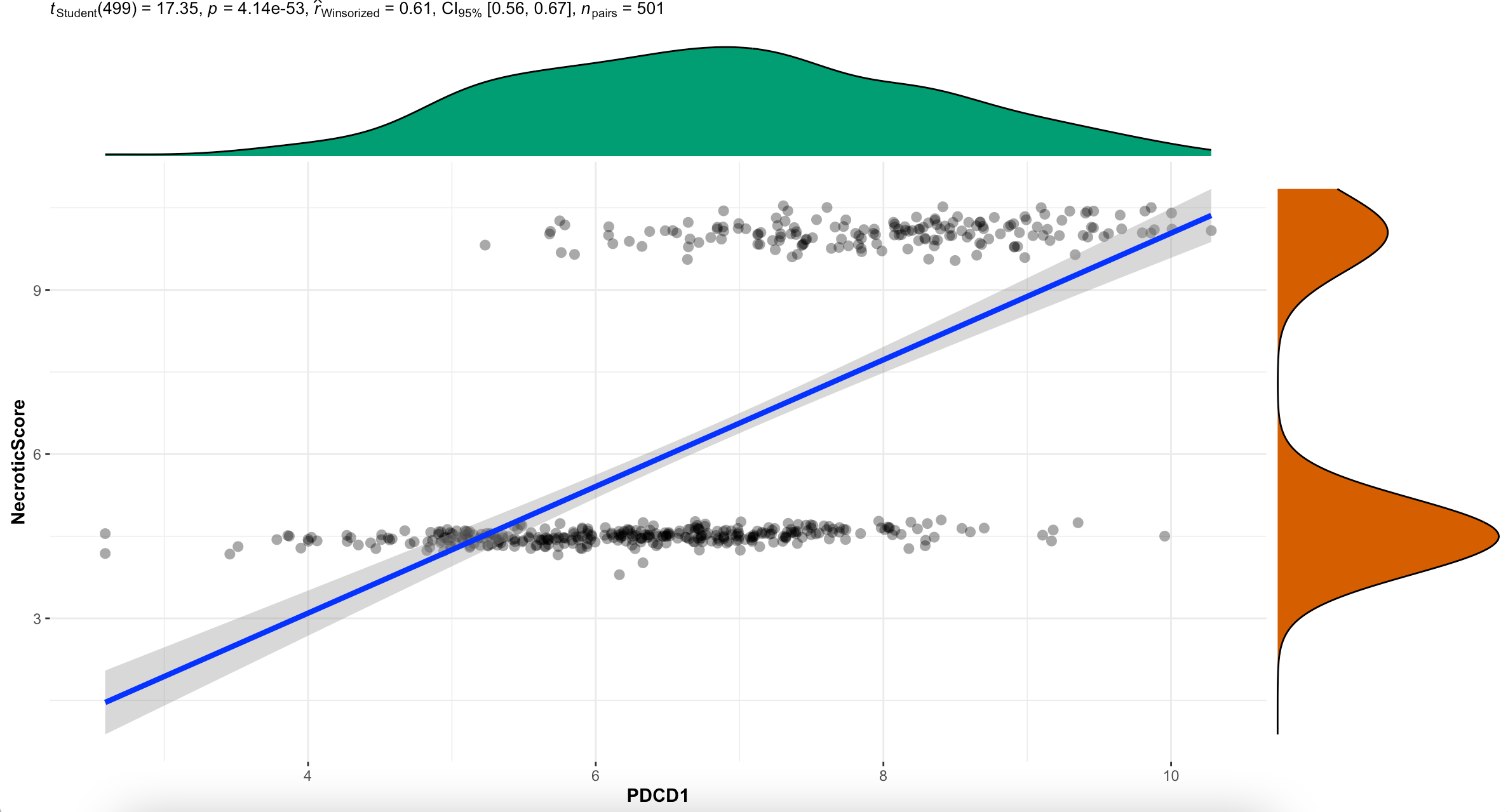

Supplement: Supplementary file 1 [file DataSheet1.ZIP › t c ga/WechatIMG49.png]

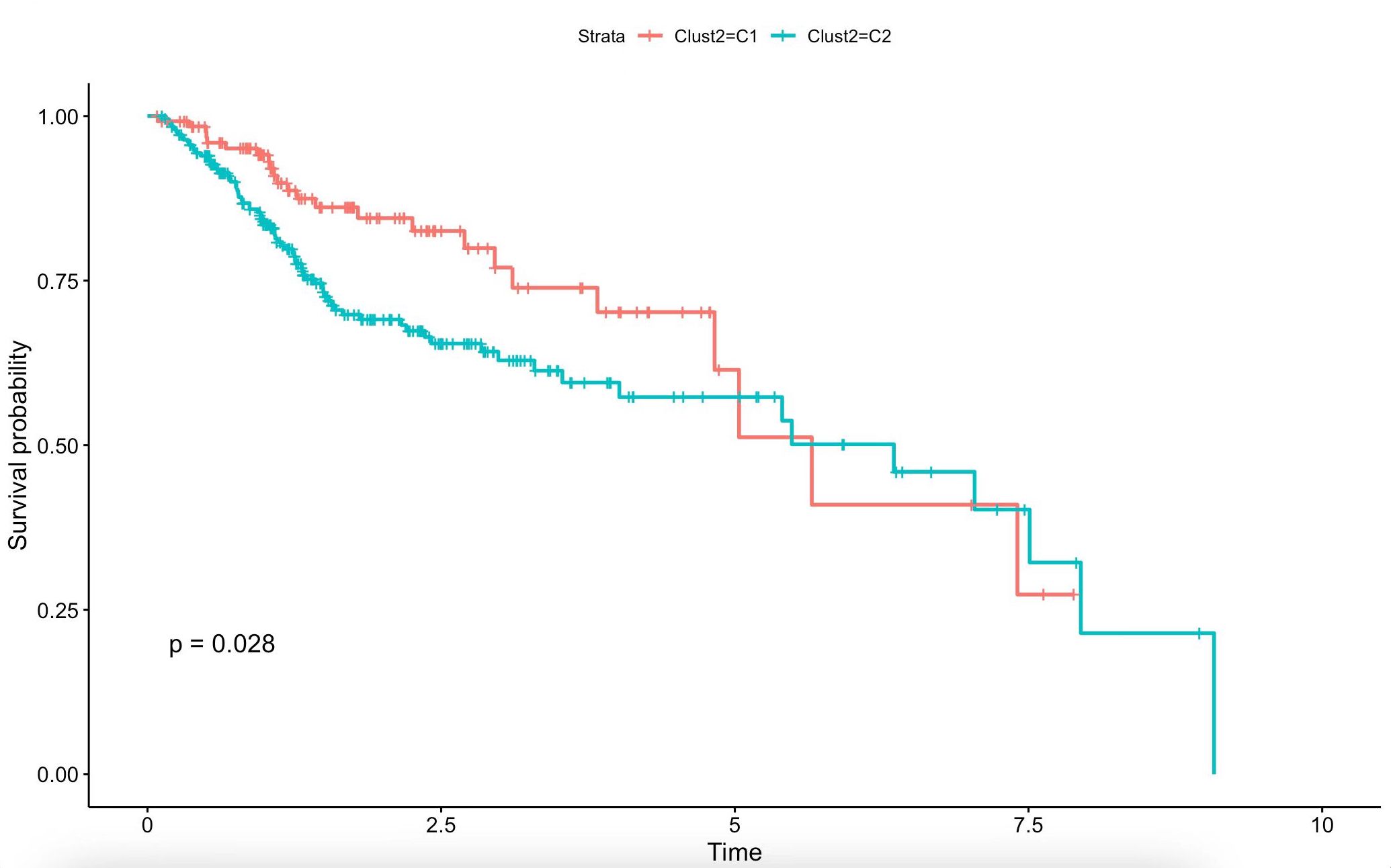

Supplement: Supplementary file 1 [file DataSheet1.ZIP › t c ga/WechatIMG27.jpeg]

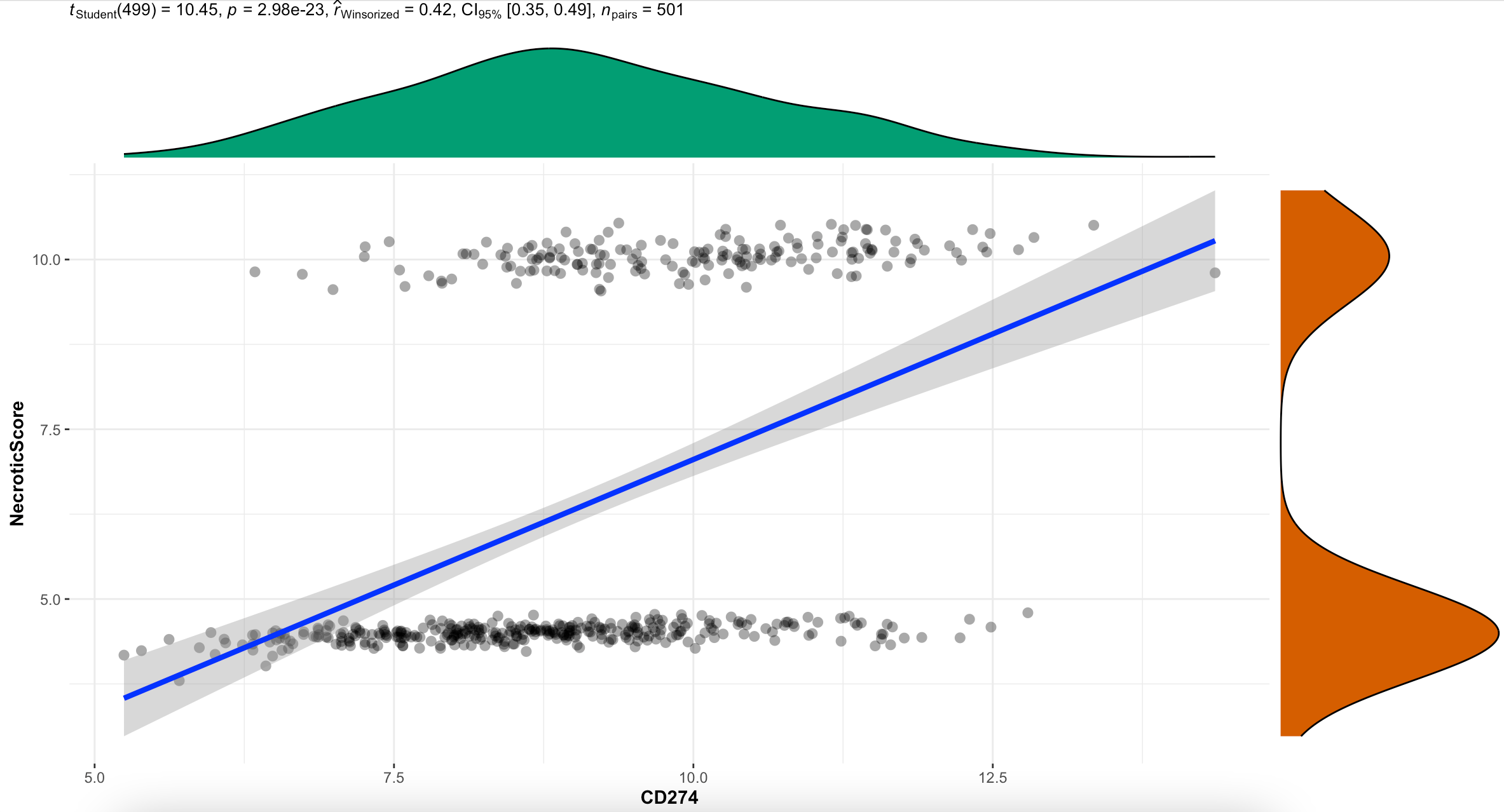

Supplement: Supplementary file 1 [file DataSheet1.ZIP › t c ga/WechatIMG48.png]

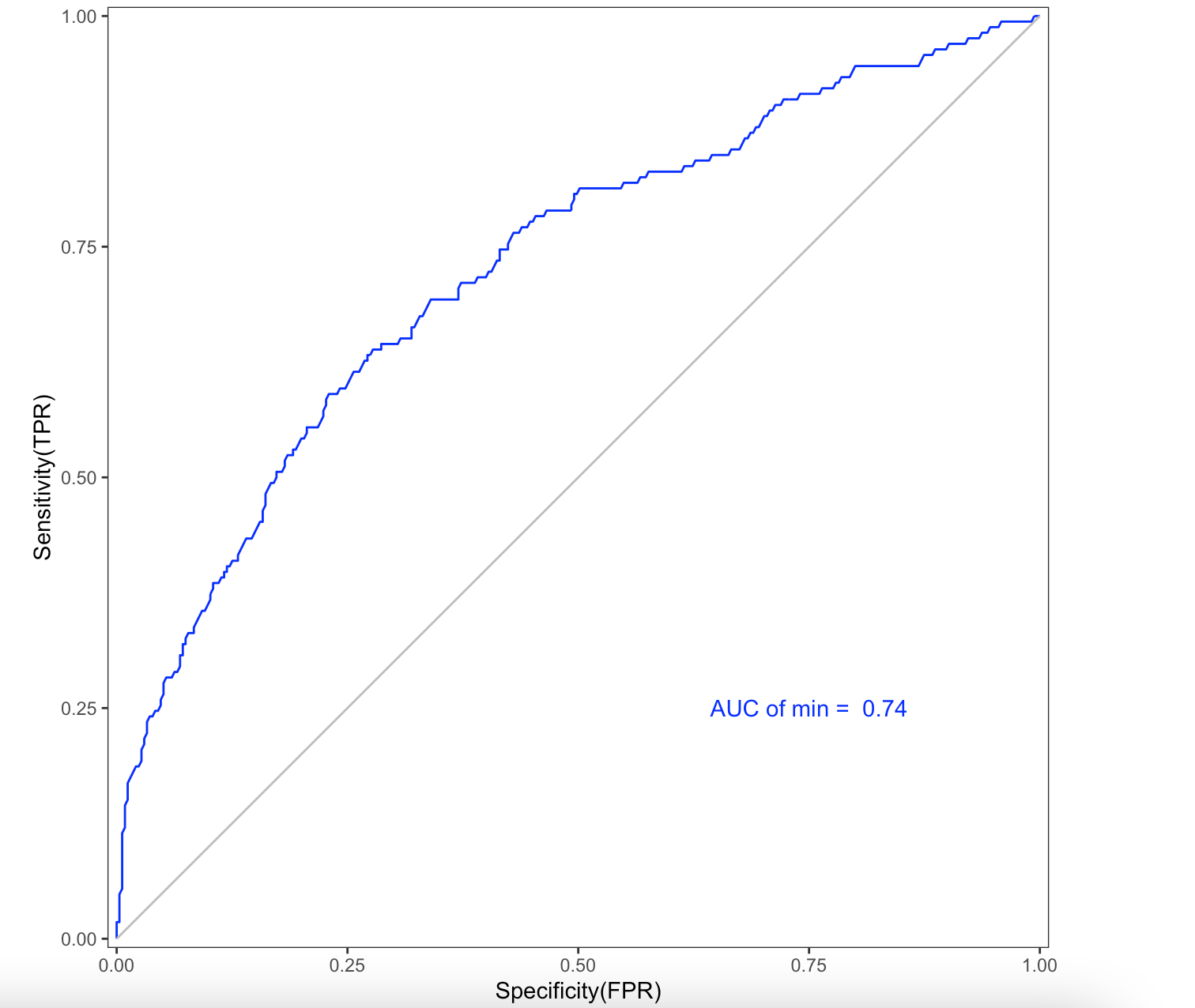

Supplement: Supplementary file 1 [file DataSheet1.ZIP › t c ga/WechatIMG60.png]

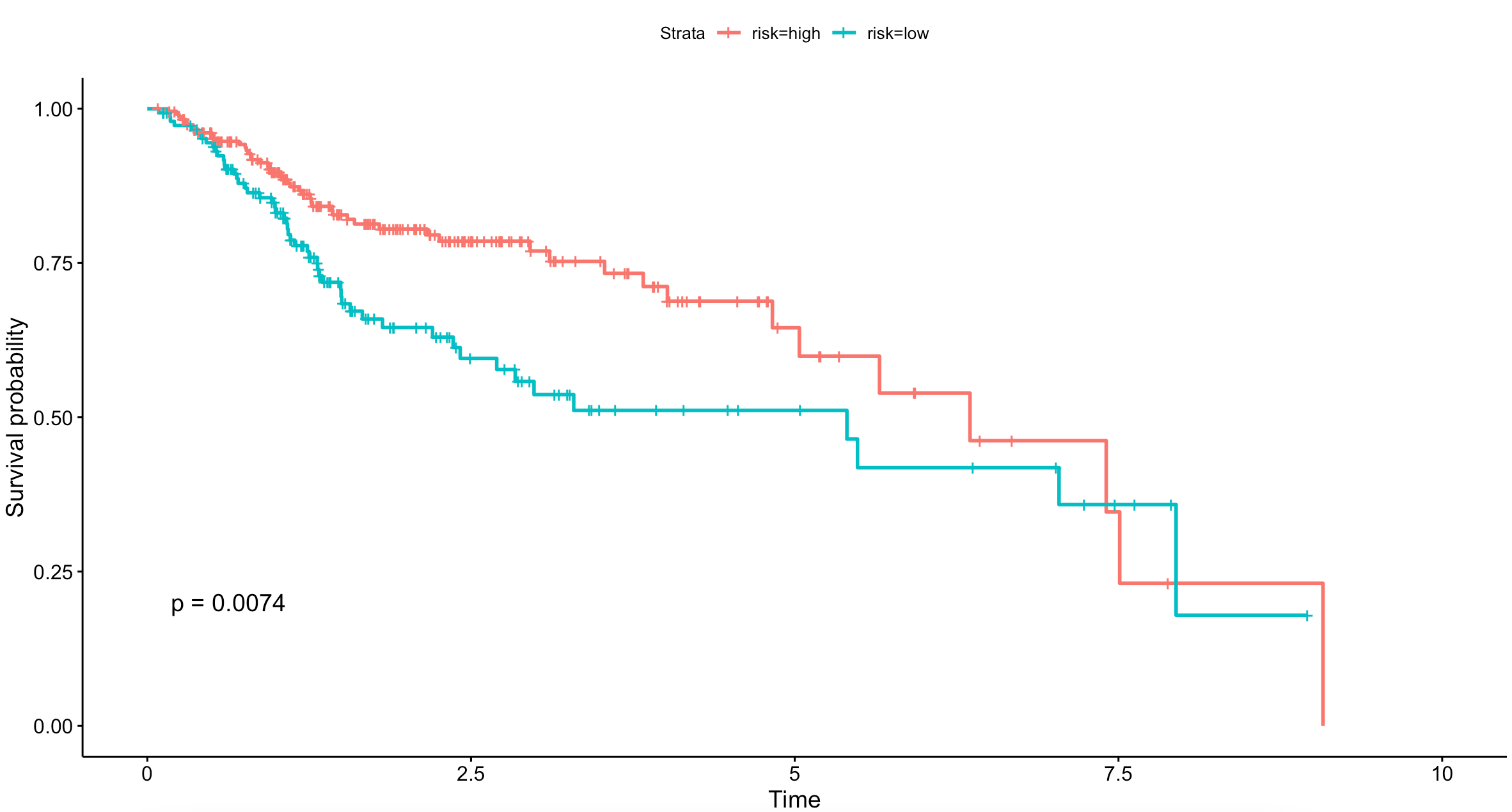

Supplement: Supplementary file 1 [file DataSheet1.ZIP › t c ga/WechatIMG62.png]

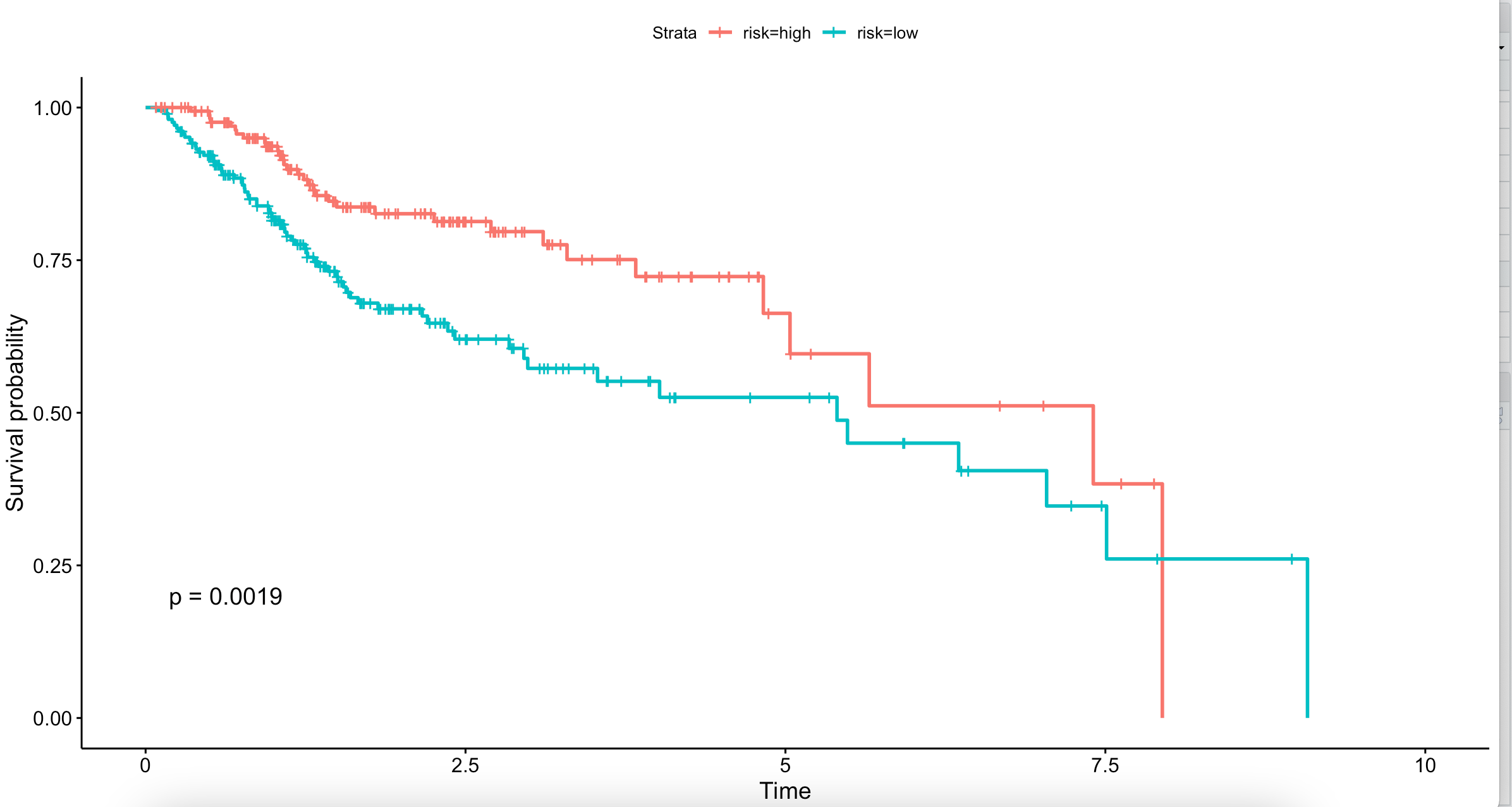

Supplement: Supplementary file 1 [file DataSheet1.ZIP › t c ga/WechatIMG63.png]

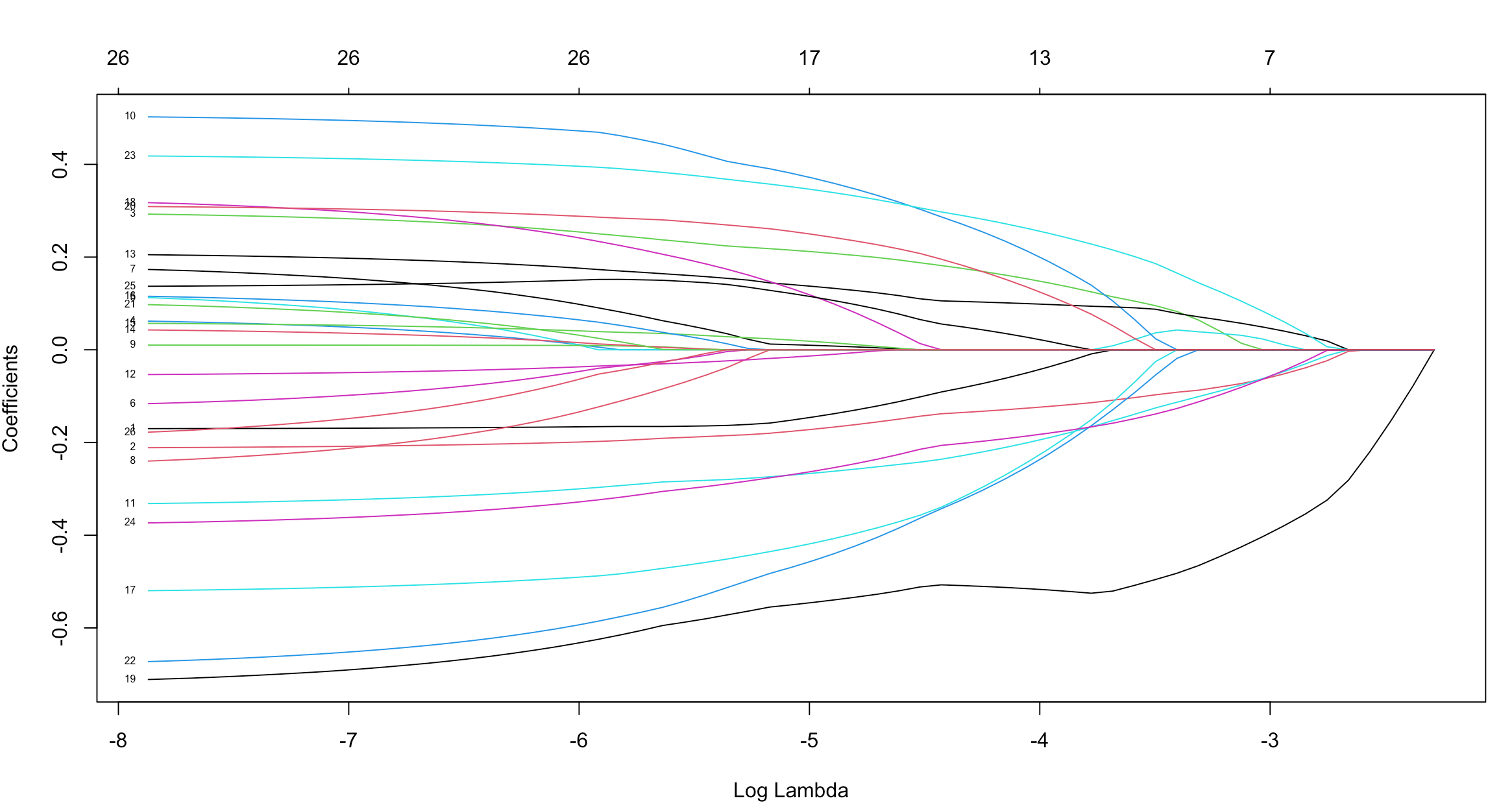

Supplement: Supplementary file 1 [file DataSheet1.ZIP › t c ga/WechatIMG58.png]

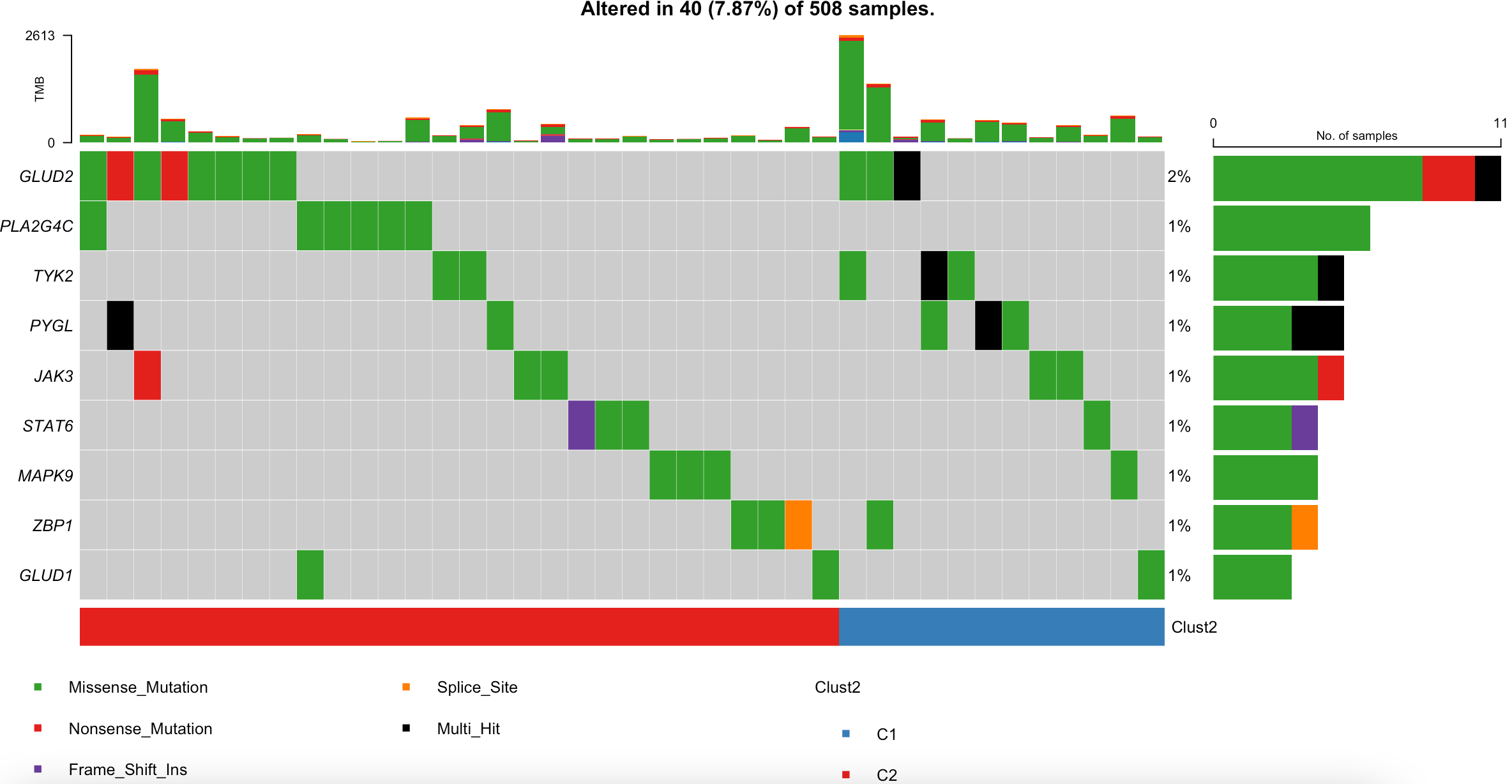

Supplement: Supplementary file 1 [file DataSheet1.ZIP › t c ga/WechatIMG64.png]

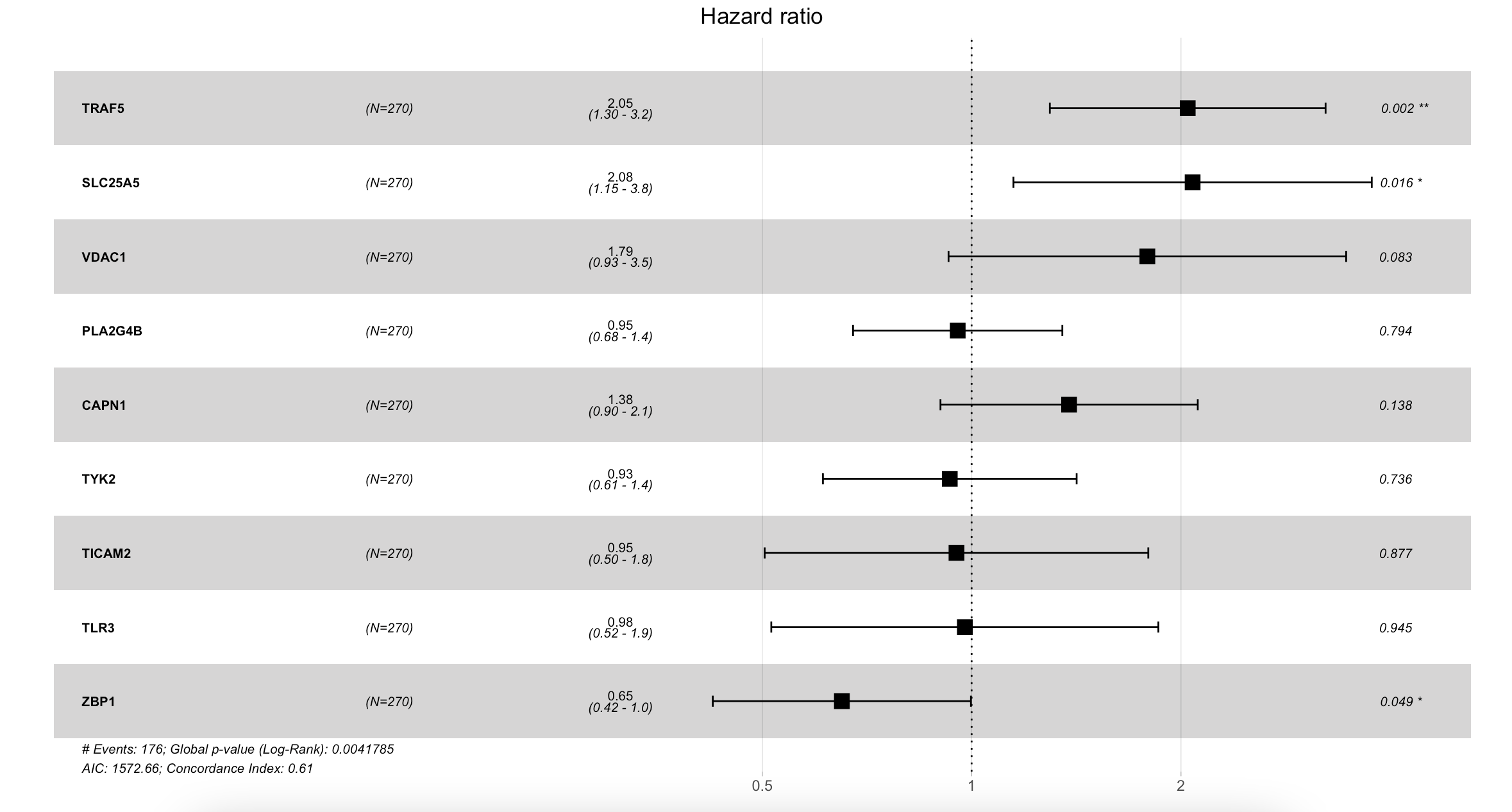

Supplement: Supplementary file 3 [file DataSheet2.ZIP › gse65858/WechatIMG41.png]

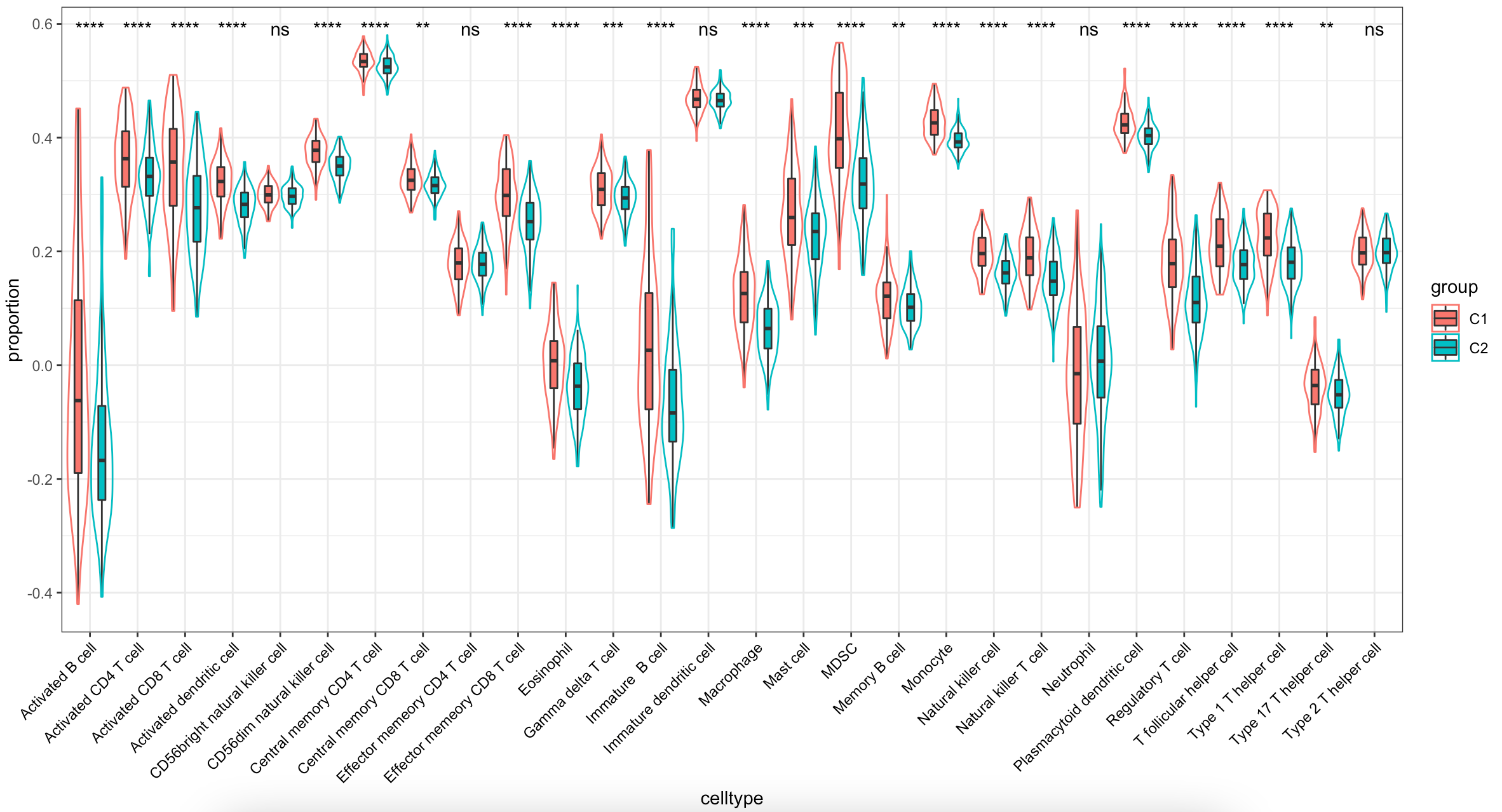

Supplement: Supplementary file 3 [file DataSheet2.ZIP › gse65858/WechatIMG46.png]

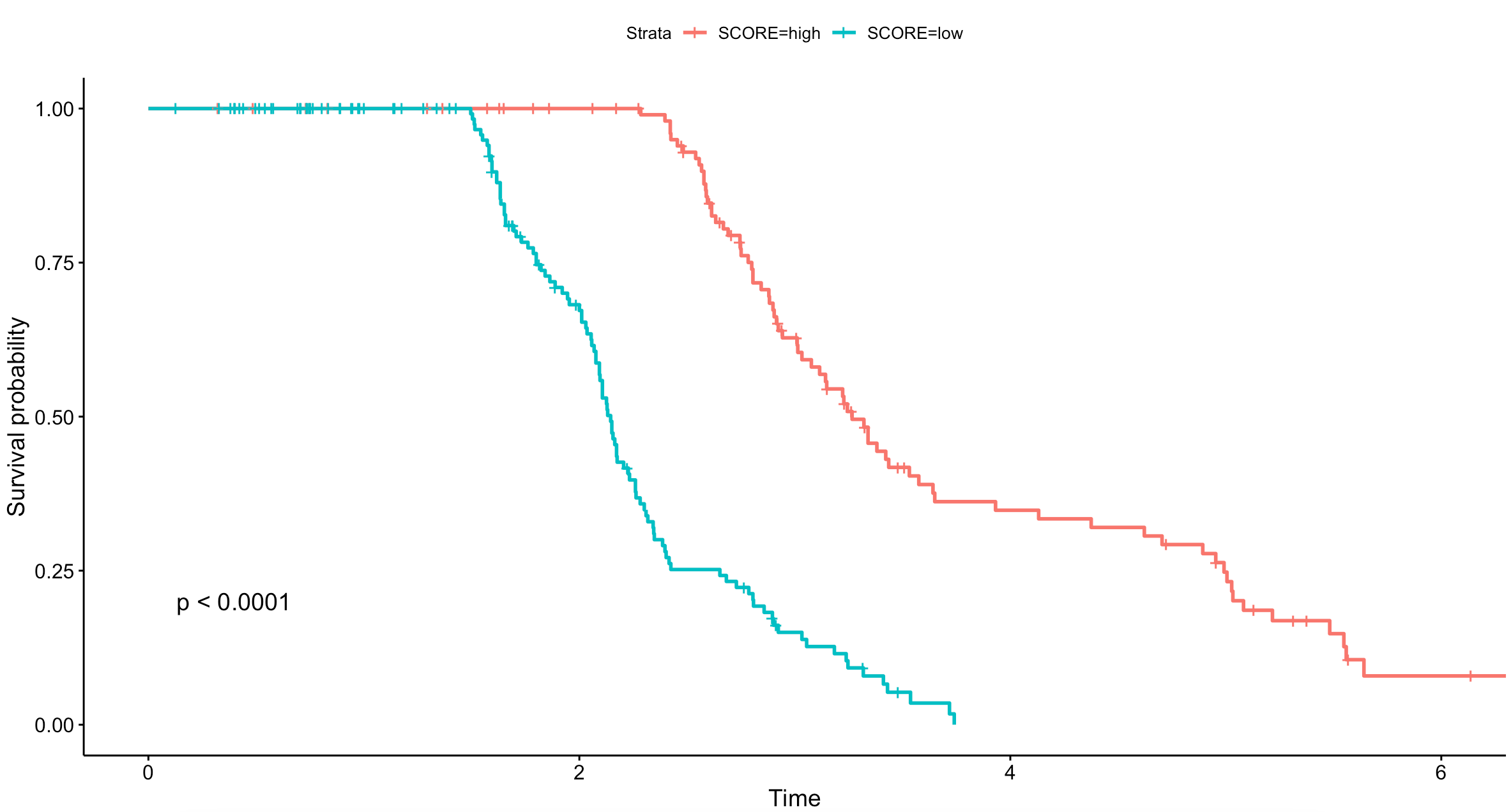

Supplement: Supplementary file 3 [file DataSheet2.ZIP › gse65858/WechatIMG34.png]

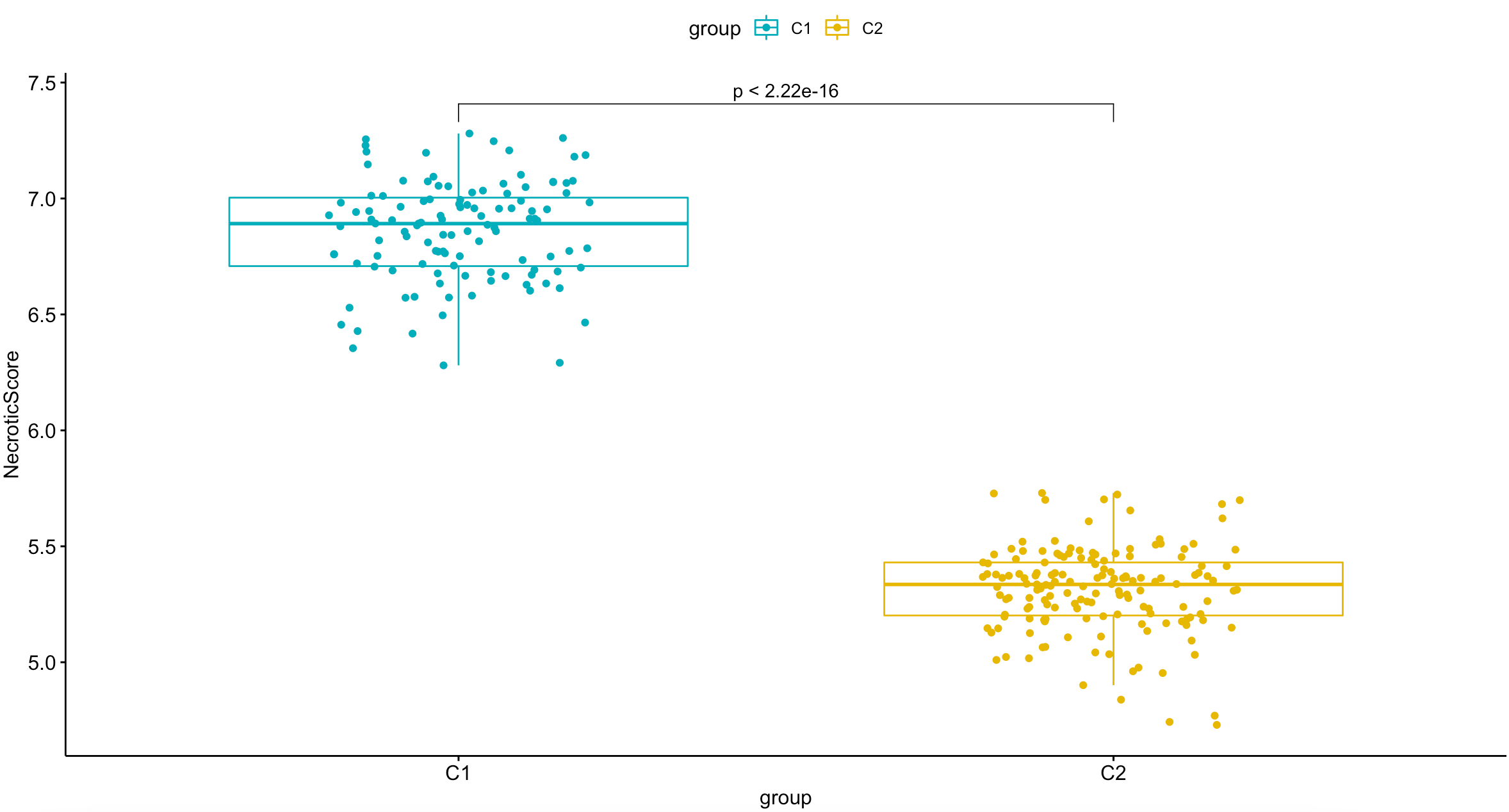

Supplement: Supplementary file 3 [file DataSheet2.ZIP › gse65858/WechatIMG35.png]

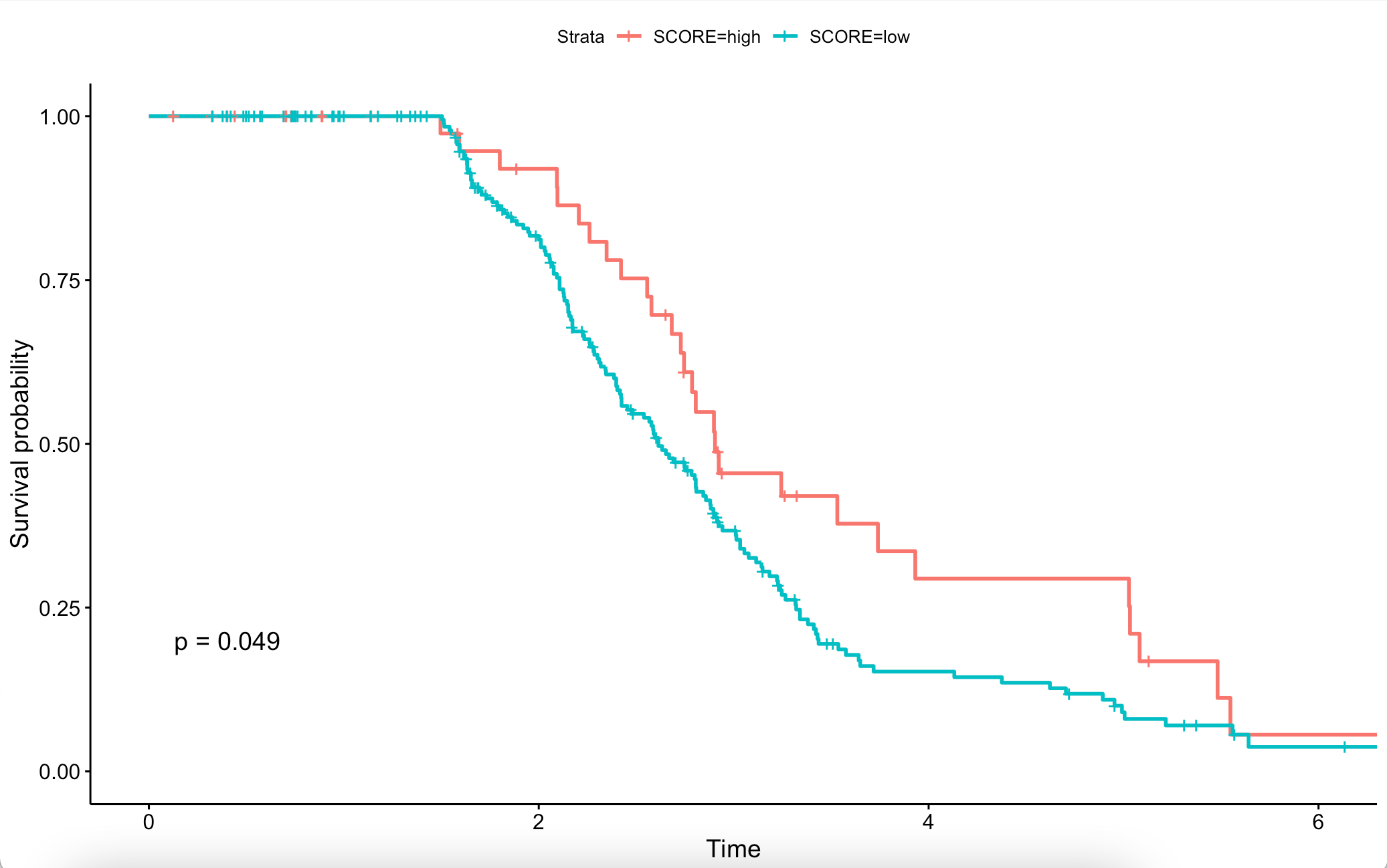

Supplement: Supplementary file 3 [file DataSheet2.ZIP › gse65858/WechatIMG24.png]

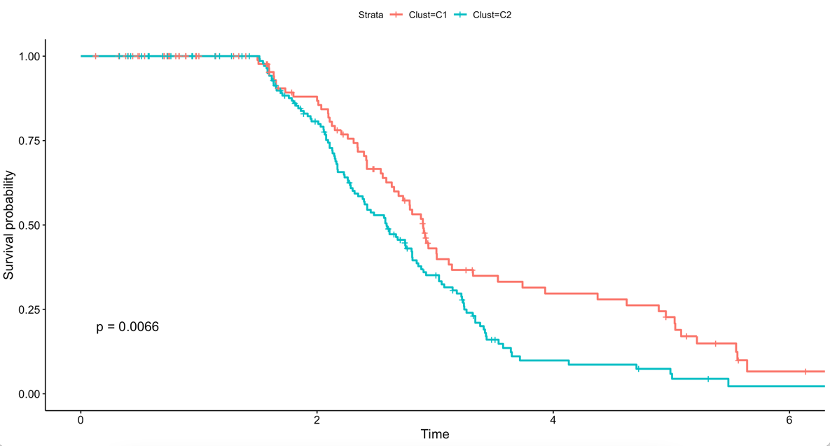

Supplement: Supplementary file 3 [file DataSheet2.ZIP › gse65858/WechatIMG33.png]

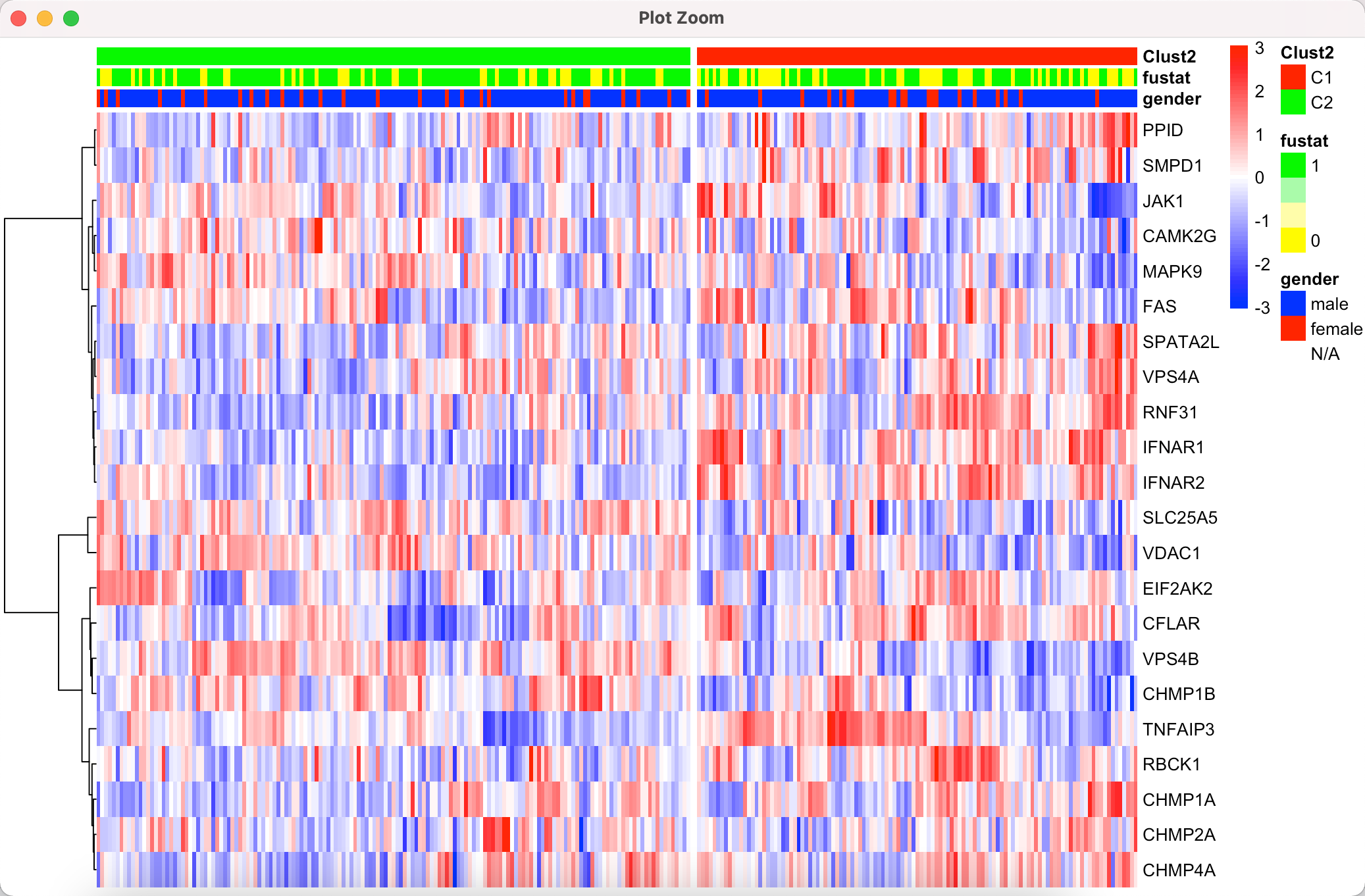

Supplement: Supplementary file 3 [file DataSheet2.ZIP › gse65858/WechatIMG14.png]

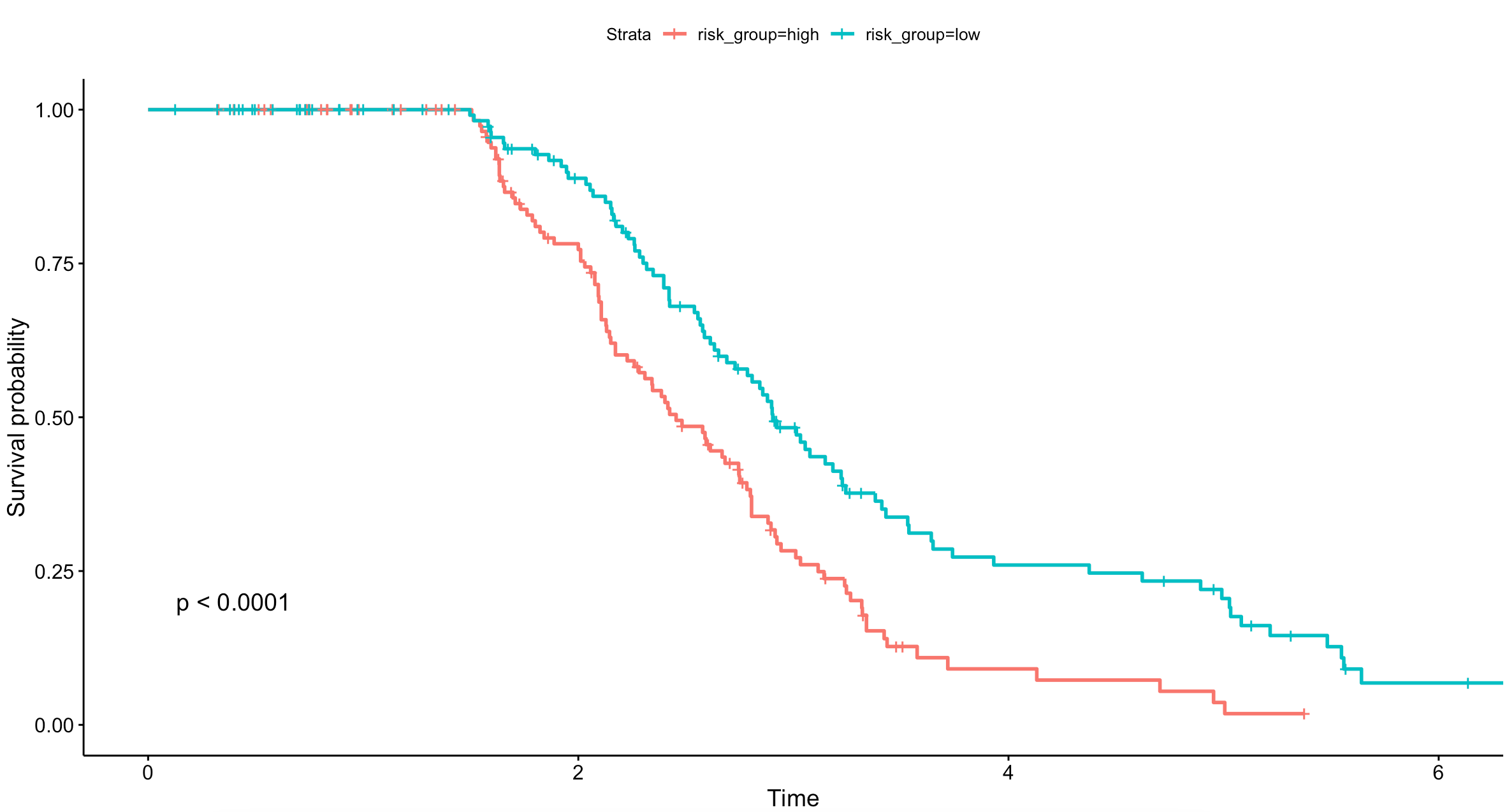

Supplement: Supplementary file 3 [file DataSheet2.ZIP › gse65858/WechatIMG48.png]
