# Supplementary material for: Necroptosis is Related to Anti-PD-1 Treatment Response and Influences the Tumor Microenvironment in Head and Neck Squamous Cell Carcinoma
Source: Front Genet. 2022 May 25;13:862143. doi: 10.3389/fgene.2022.862143 (PMC9174803; doi:10.3389/fgene.2022.862143)
Supplement: Supplementary file 2 [file Table1.DOC]

**Supplementary table 1**

|  | **Dead**  **(N=335)** | **Alive**  **(N=166)** | **p-value** |
| --- | --- | --- | --- |
| **Gender(n,%)** |  |  | 0.0699 |
| female | 80(23.9) | 53(31.9) |  |
| male | 255(76.1) | 113(68.1) |  |
| **HPV(n,%)** |  |  | 0.316 |
| indeterminate | 2(0.6) | 0(0) |  |
| negative | 269(80.3) | 141(84.9) |  |
| positive | 64(19.1) | 25(15.1) |  |
| **Event (n,%)** |  |  | <0.001 |
| 0 | 335(100) | 0(0) |  |
| 1 | 0(0) | 166(100) |  |
| **Stage (n,%)** |  |  | 0.0677 |
| i | 14(4.2) | 5(3.0) |  |
| ii | 60(17.9) | 35(21.1) |  |
| iii | 67(20.0) | 36(21.7) |  |
| iva | 173(51.6) | 82(49.4) |  |
| ivb | 5(1.5) | 4(2.4) |  |
| ivc | 2(0.6) | 4(2.4) |  |
| NA | 14(4.2) | 0(0) |  |
